# Dual color pH probes made from silica and polystyrene nanoparticles and their performance in cell studies

**DOI:** 10.1038/s41598-023-28203-0

**Published:** 2023-01-24

**Authors:** Priyanka Srivastava, Isabella Tavernaro, Lena Scholtz, Claudia Genger, Pia Welker, Frank Schreiber, Klas Meyer, Ute Resch-Genger

**Affiliations:** 1 Division Biophotonics, Federal Institute for Materials Research and Testing (BAM), Richard-Willstaetter-Str. 11, 12489 Berlin, Germany; 2 Institut für Chemie und Biochemie, Freie Universität Berlin, Takustr. 3, 14195 Berlin, Germany; 3 nanoPET Pharma GmbH, Robert‐Koch‐Platz 4, 10115 Berlin, Germany; 4 Charité-Universitätsmedizin Berlin, Charitéplatz 1, 10117 Berlin, Germany; 5 Division Biodeterioration and Reference Organisms, Federal Institute for Materials Research and Testing (BAM), Unter den Eichen 87, 12205 Berlin, Germany; 6 Division Process Analytical Technology, Federal Institute for Materials Research and Testing (BAM), Richard-Willstaetter-Str. 11, 12489 Berlin, Germany

**Keywords:** Nanoparticles, Biophysics

## Abstract

Ratiometric green–red fluorescent nanosensors for fluorometrically monitoring pH in the acidic range were designed from 80 nm-sized polystyrene (PS) and silica (SiO_2_) nanoparticles (NPs), red emissive reference dyes, and a green emissive naphthalimide pH probe, analytically and spectroscopically characterized, and compared regarding their sensing performance in aqueous dispersion and in cellular uptake studies. Preparation of these optical probes, which are excitable by 405 nm laser or LED light sources, involved the encapsulation of the pH-inert red-fluorescent dye Nile Red (NR) in the core of self-made carboxylated PSNPs by a simple swelling procedure and the fabrication of rhodamine B (RhB)-stained SiO_2_-NPs from a silane derivative of pH-insensitive RhB. Subsequently, the custom-made naphthalimide pH probe, that utilizes a protonation-controlled photoinduced electron transfer process, was covalently attached to the carboxylic acid groups at the surface of both types of NPs. Fluorescence microscopy studies with the molecular and nanoscale optical probes and A549 lung cancer cells confirmed the cellular uptake of all probes and their penetration into acidic cell compartments, i.e., the lysosomes, indicated by the switching ON of the green naphthalimide fluorescence. This underlines their suitability for intracellular pH sensing, with the SiO_2_-based nanosensor revealing the best performance regarding uptake speed and stability.

## Introduction

One of the most frequently measured environmental parameters in the life sciences using fluorescent probes is pH^1–7^. This is related to its importance for proper cell function and its potential to act as an indicator for inflammatory diseases and cancer. Although the pH value in the cytoplasm of tumour cells is not very different to healthy cells, the pH value of their microenvironment is significantly altered and is a potential target for cancer therapy^8^. Moreover, the determination and monitoring of pH values in different cell compartments allows, e.g., to localize vesicular structures of the endosomal-lysosomal apparatus in cells. The lowest pH values of about 4.5 are reached in the lysosomes involved in autophagy, protein degradation, apoptosis, and cell defence mechanism^9,10^. The interest in measuring pH in biological systems together with the general advantages offered by fluorescence methods such as relatively simple and inexpensive instrumentation^11,12^, minimal invasiveness, and suitability for the in situ online monitoring of local pH using, e.g., optical microscopy, and remote sensing^11,12^ has triggered the development of pH-responsive organic fluorophores from different dye classes. This includes BODIPY and BF_2_-chelated tetraarylazadipyrromethene dyes^13–18^, as well as xanthene^19–24^, cyanine^25–29^, squaraine^23^, and naphthalimide fluorophores^30–33^, which exploit intramolecular photoinduced electron transfer (PET), intramolecular charge transfer (ICT), and fluorescence energy transfer processes for the signaling of pH. Optical parameters utilized for the determination and monitoring of pH are pH-induced changes in the spectral position and/or intensity of the dye’s absorption and/or emission bands or fluorescence lifetime^31,34–37^. In addition, protonation-induced ring opening and closing mechanisms accompanied by characteristic modifications in the absorption and emission properties have been employed for pH sensing^3,38^.

In the last decades, also an increasing number of modularly built nanoparticle (NP)-based sensors has been reported, utilizing, e.g., silica and polymer NPs, lanthanide-based upconversion NPs, and semiconductor quantum dots in combination with organic sensor dyes^4,19,39–46^. Nanomaterials which have been the most frequently employed for the preparation of nanosensors for bioimaging applications are organic polymeric particles such as biocompatible polystyrene (PS) NPs (PSNPs), and inorganic silica (SiO_2_) NPs (SiO_2_-NPs) that can be prepared in a variety of sizes with different surface functionalities^47–51^. Advantages of dye-based polymer and silica nanosensors compared to molecular sensors are a straightforward signal amplification, the possibility to apply hydrophobic fluorophores for analyte sensing in aqueous environments, and the ease of combining two or more different fluorophores for realizing ratiometric or self-referenced sensors^52^, which read out the quotient of a spectrally distinguishable analyte responsive and an analyte insensitive (reference) fluorescence signal at a single excitation wavelength. With this sensor design, fluctuations of the excitation light source can be elegantly considered, which would otherwise directly affect and distort the fluorescence output of the sensor^52,53^. In the case of polymer and silica nanomaterials, ratiometric nanosensors are commonly obtained by either incorporating a sensor dye and a reference dye into the particle core or by the staining of the particle core with a reference dye followed by the covalent attachment of the sensor molecules to functional groups at the particle surface^19,54^. These surface groups can be also utilized for the binding of recognition moieties such as (bio)molecules, proteins, and antibodies^55,56^, yielding targeted nanostructures. Core encapsulation enables the utilization of hydrophobic fluorophores without reactive groups and can increase the stability particularly of near infrared (NIR)-emissive chromophores. For sensor dyes, core encapsulation is only suitable if the target analyte can penetrate the particle matrix. In the case of polymer particles such as PSNPs, core staining with hydrophobic organic dyes can be easily achieved with a simple swelling procedure, utilizing premanufactured NPs that bear surface functionalities such as carboxyl or amino groups^57,58^. SiO_2_-NPs can be encoded with dyes by covalently coupling reactive dyes to the silica precursors, forming the silica matrix of the core or shells surrounding the core. Alternatively, physisorption during PSNP or SiO_2_-NP formation can be exploited, thereby sterically incorporating the dye molecules into the polymer or silica network. This requires fluorophores that are sufficiently stable under the reaction conditions employed for NP formation.

In the following, we present a comparative screening study of the sensing potential of two sets of closely matching ratiometric pH nanosensors made from PSNPs and SiO_2_-NPs of comparable size which were core stained with a red emissive reference dye and surface-functionalized with a novel hydrophilic neutral PET-operated naphthalimide dye that exhibits a pH-responsive green fluorescence. This includes the synthesis and spectroscopic characterization of the pH-sensitive naphthalimide dye and the analytical and optical characterization of the different nanosensors, providing application-relevant particle properties such as size, size distribution, surface charge / zeta potential, sensor dye labeling density, and fluorescence quantum yields, as well as reversibility and stability studies. Subsequently, cellular uptake and imaging studies were performed with both types of nanosensors. As we aimed for a comparison of both types of nanosensors and not a complete characterization of their sensor parameters and performance, we kept as many nanosensor properties as closely matching as possible including particle size, sensor dye, spectroscopic properties of the reference dyes, nanosensor surface chemistry, and number of surface-bound sensor dyes. In addition, all experiments were performed with the same NP concentration to enable a comparison of the pH sensing potential of the polymer and silica nanosensors.

## Results and discussion

**Figure 1:**
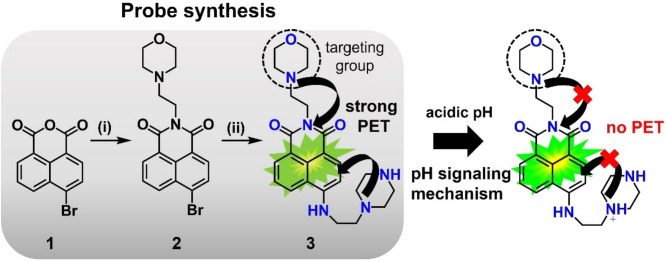
Synthesis of the hydrophilic neutral optical pH probe **3** designed for lysosomal targeting and its pH signaling mechanism; (i) 4-(2-Aminoethyl) morpholine, ethanol, 50 °C,5 h; (ii) 1-(2-Aminoethyl) piperazine, triethylamine, pyridine, reflux, overnight. The pH signaling mechanism of **3** relies on an intramolecular photoinduced electron transfer (PET) from the unprotonated nitrogen atoms of the piperazine and morpholine units to the naphthalimide chromophore. Nitrogen protonation results in the blocking of these fluorescence quenching PET processes leading to the appearance of the chromophore’s green fluorescence at acidic pH values as confirmed in the following sections.

As a prerequisite for the fabrication of green–red emissive pH nanosensors utilizing custom made PSNPs and SiO_2_-NPs, we developed the hydrophilic PET-operated, naphthalimide-based fluorescent pH probe **3** shown in Fig. 1, intended for the signaling of acidic pH values by the protonation-induced switching ON of its green fluorescence and allow for lysosomal targeting^31,59,60^. Compared to other pH sensitive dyes such as fluorescein, the incorporated lysosomal targeting morpholine group was expected to be beneficial for the intended cell studies. The synthesis of optical probe **3**, which is summarized in Fig. 1 and detailed in the Supporting Information (SI; Figs. S1–S10), involved the covalent linking of a morpholine unit to 4-bromo-1,8-naphthalic anhydride **1** for the selective lysosomal targeting and the incorporation of a piperazine moiety to improve the water solubility and induce a pH sensitivity of the chromophore’s fluorescence. Subsequently, the optical properties of **3** were characterized by absorption and emission spectroscopy and fluorescence quantum yield measurements at different pH values.

**Figure 2:**
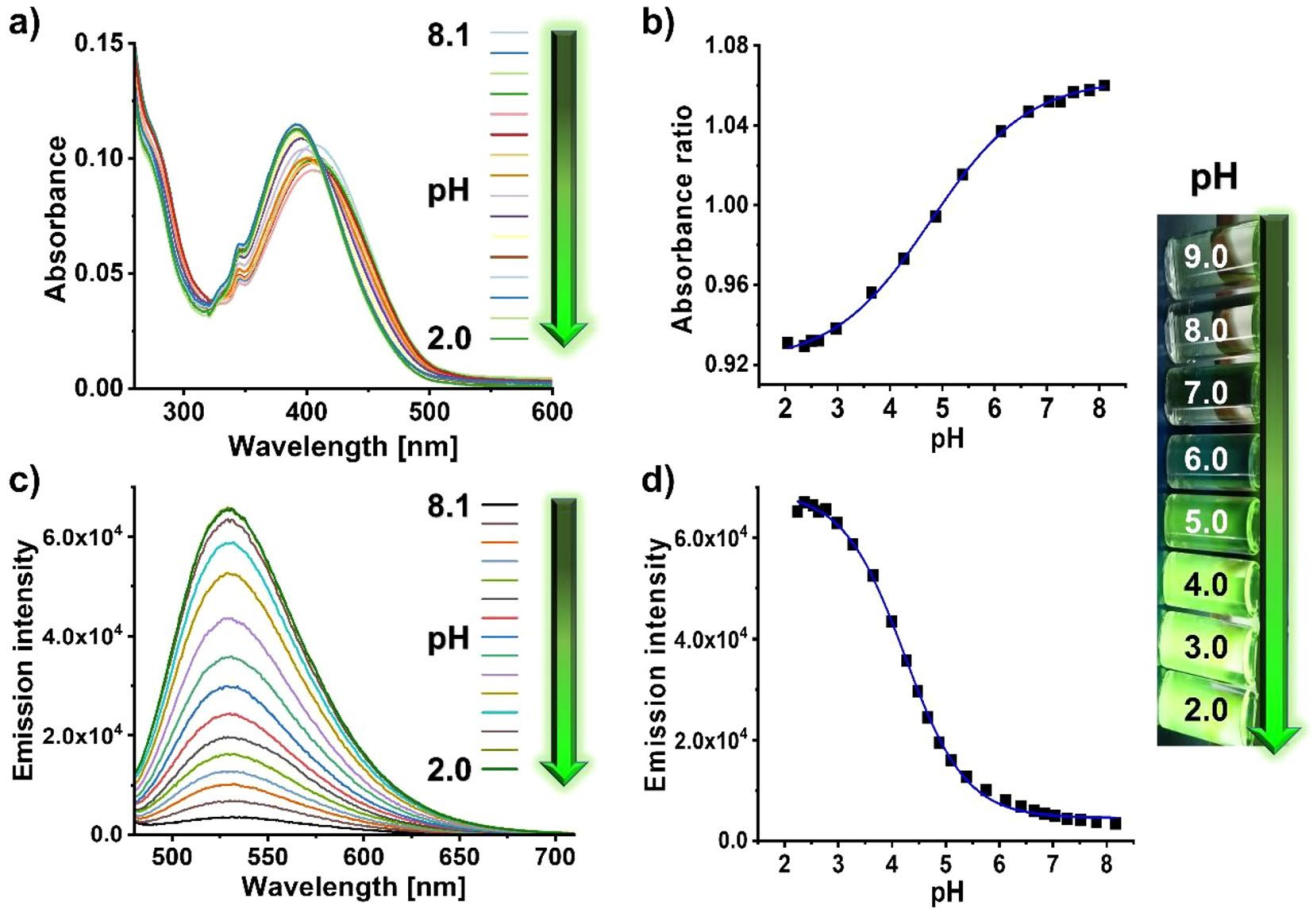
pH-dependent spectroscopic properties of fluorescent probe **3**; (**a**) absorption spectra (25 µM) at different pH values; (**b**) plot of the absorbance ratio (405 nm/390 nm) at different pH values; (**c**) emission spectra (0.13 µM) at different pH values; and (**d**) emission intensity (λ_Ex_ = 405 nm) at different pH values used to calculate the pK_a_ of the optical probe. The inset shows a photograph of the color changes of the emission of **3** under UV light at different pH values that can be detected by naked eye.

As shown in Fig. 2, fluorescent probe **3** has a broad absorption band at 405 nm (ε = 4,920 M^−1^ cm^−1^) that is attributed to an ICT from the unprotonated nitrogen atoms of the piperazine and morpholine units to the naphthalimide chromophore. **3** exhibits a very weak emission centered at 530 nm upon excitation (λ_Ex_) at 405 nm in Britton Robinson (B–R) buffer at a pH of 8.1. The low fluorescence intensity at neutral and basic pH values is ascribed to PET-induced quenching of the naphthalimide fluorescence by the nitrogen atoms of the morpholine and piperazine moiety, that can be reversibly blocked at acidic pH values as was observed for other naphthalimide-based fluorescent probes^30–33^. The ICT and PET processes controlling the photophysics of **3** were confirmed by nuclear magnetic resonance spectrometry (NMR) measurements in D_2_O in the pH range of 8.5 to 2.5 (SI, Fig. S11). As revealed by this NMR study, the amine group at the 4th position acts as a donor and the naphthalimide chromophore as an acceptor for the ICT process determining the absorption features of **3**. Protonation of the nitrogen atoms of the morpholine and piperazine moiety at acidic pH values, indicated by a downfield shift of the aromatic and some aliphatic signals of the morpholine and piperazine unit in the NMR spectrum, prevents PET, thereby turning ON the green naphthalimide fluorescence. This provides the basis for the pH-dependent reversible ON–OFF switching of probe **3**.

A more detailed spectroscopic study of the pH sensing behavior of **3** in the pH range of 8.1 to 2.0 in B-R buffer revealed a hypsochromic shift of the ICT absorption band from 405 to 392 nm for pH < 5 and a decrease in absorbance for further decreasing pH values (Fig. 2 and SI, Fig. S12). These pH-induced spectral and intensity changes in absorption were subsequently considered for the calculation of the probe’s pK_a_ and the fluorescence measurements. Fluorescence measurements confirmed a gradual increase in the intensity of the naphthalimide fluorescence at 530 nm that remained eventually constant at a pH value of 2.5. A sigmoidal fit of the plot of the pH-dependent fluorescence intensity at 530 nm gave a p*K*_*a*_ = 4.23 ± 0.02. The pKa value of **3** is suitable for lysosomal targeting as the lysosomes exhibit pH values of about 4.5^61^. In the inset of Fig. 2, the visual changes in the emission color under ultraviolet (UV) light in the pH range of 9.0 (colorless) to 2.0 (green) are displayed that can be detected by naked eye. The fluorescence quantum yield of **3**, which was absolutely measured in B-R buffer in the pH range of 8.1 to 2.0, revealed a constant increase for decreasing pH values (SI, Fig. S13 and Table S1) and amounted to 37% for fully protonated probe **3**.

### Reversibility and photostability studies with optical probe 3

Reversibility studies with **3** performed at pH values varied between 8.1 and 4.8 showed no loss in fluorescence. This is a prerequisite for the applicability of **3** as a pH sensor (SI, Fig. S14). Photostability studies of **3** at pH 4.0 in B-R buffer at an illumination wavelength of λ_Ex_ = 405 nm utilizing the 450 W xenon lamp of the spectrofluorometer revealed only small changes in the fluorescence spectrum and fluorescence intensity of **3** for illumination times of up to 8 h (SI, Fig. S15).

### Dual color emissive ratiometric PSNP- and SiO_2_-NP based nanosensors

**Figure 3:**
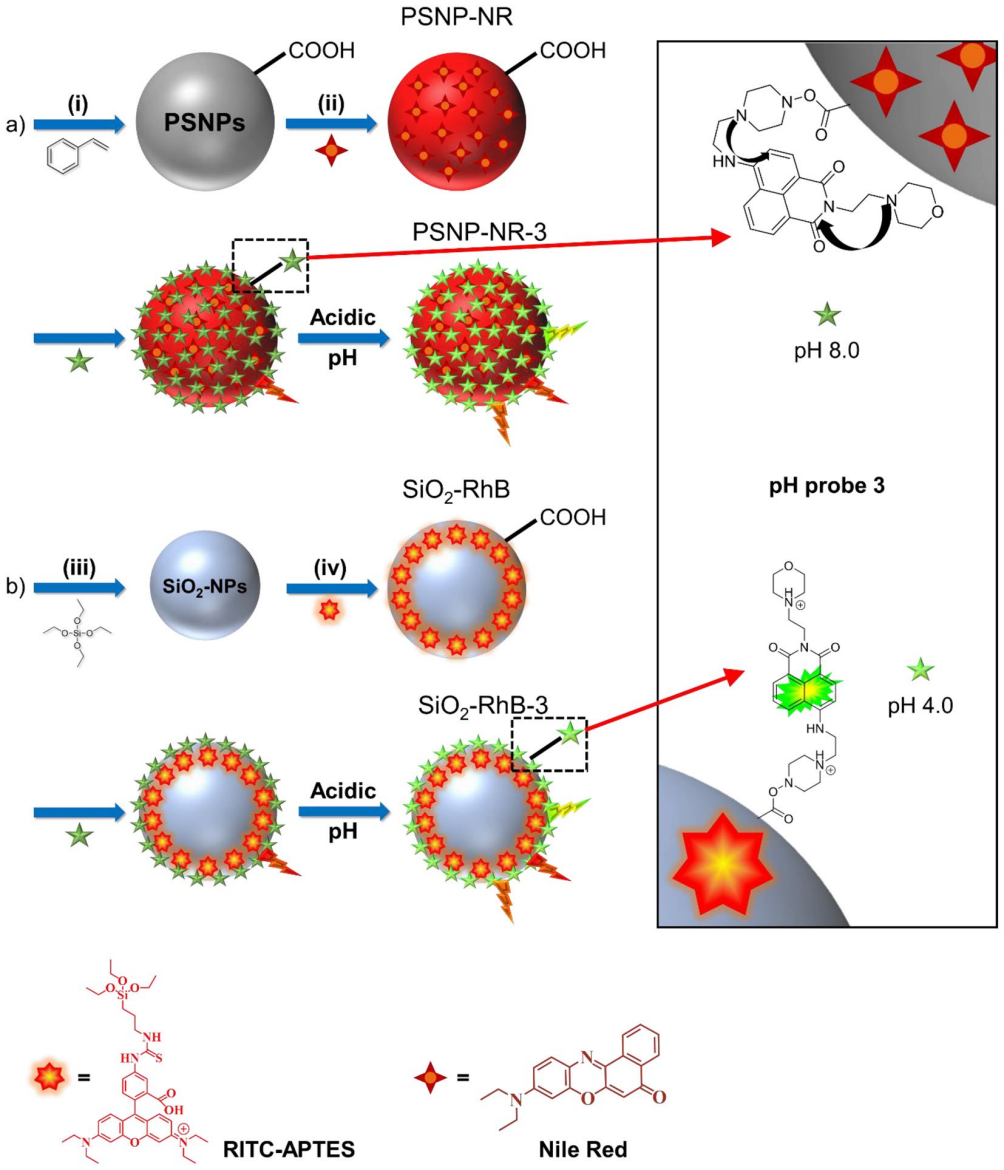
(**a**) Schematic representation of the synthesis of Nile Red (NR) loaded PSNPs, where the pH-inert reference dye is homogeneously distributed within the particle core; (i) synthesis of pristine PSNP-COOH, first step: SDS, PPS, MilliQ-H_2_O, 70 °C, 1 h, second step: AA, MilliQ-H_2_O, 70 °C, 3 h, (ii) NR loading by swelling procedure, NR, THF, room temperature (r.t.), 30 min; (**b**) SiO_2_-NP synthesis and staining with a self-made pH-inert rhodamine B (RhB)-silane derivative, (iii) cyclohexane/MilliQ-water, l-arginine, 60 °C, 20 h (1 × seed growing, 2 × regrowth steps); (iv) first step: RhB-APTES, cyclohexane/MilliQ-water, l-arginine, 60 °C, 20 h, second step: APTES, EtOH, Ar, r.t., 20 h, third step: succinic anhydride, DMF, Ar, 40 °C, 20 h. The reference dye is located in the outer silica shell surrounding the unstained silica core. The final NR- and RhB-loaded PSNPs and SiO_2_-NPs bear carboxylic acid groups on the particle surface introduced in one step (PSNP-COOH) and two step grafting reactions (SiO_2_-NP-COOH). Surface modification of both types of carboxylated NPs with optical probe **3** (green star) using EDC/NHS coupling chemistry yielded the green–red emissive ratiometric nanosensors PSNP-NR-**3** and SiO_2_-RhB-**3**.

In the next step, we prepared two sets of ratiometric pH nanosensors by combining our lysosomal targeting pH probe with dye-stained PSNPs and SiO_2_-NPs of closely matching size. Therefore, pristine carboxylated PSNPs with an average particle size of about 80 nm were synthesized by an emulsion polymerization approach^55^. For core staining, the hydrophobic and neutral dye Nile Red (NR) was chosen as pH-inert reference dye and encapsulated in the polymer matrix by a simple swelling procedure (Fig. 3a^41,57^. The synthesis of the amorphous SiO_2_-NPs of similar size involved multistep hydrolysis and condensation reactions of the silicon precursor in a biphasic cyclohexane/water system (SI, Fig. S17) and different dye staining approaches. The initially pursued attempt to also use NR as a reference dye for the SiO_2_-NPs failed because the incorporation of NR into the SiO_2_ matrix by adsorptive interactions led to an increase in particle size (SI, Fig. S21), and the dye molecules were washed out during NP purification (data not shown), as has been reported before for other dyes^62^. The synthesis of a NR silane derivative (SI, Fig. S18–S20), that should enable the covalent attachment of NR to the SiO_2_ matrix to prevent dye leaking also did not yield a higher number of encapsulated dye molecules per particle. Therefore, we modified our preparation strategy and synthesized first amorphous SiO_2_-NPs with a size of 60 nm (SI, Fig. S17) followed by the shelling of the resulting SiO_2_ core with a 20 nm thick rhodamine B isothiocyanate (RhB)-stained silica layer made from a dye-silane derivative obtained by reacting RhB with an amino silane (SI, Fig. S18b)^63,64^. With this approach, the size of both types of nanosensors could be kept identical and dye leaking was prevented (Fig. 3b). Subsequent surface functionalization of the 80 nm red emissive SiO_2_-NPs with carboxylic acid groups was achieved by a two-step post-synthetic grafting reaction (Fig. 3b). Dye leakage experiments performed after each grafting reaction showed no influence of the functional groups grafted onto the particle surface (SI, Fig. S24). Thereby, the particle surface chemistry and the conjugation chemistry used for the covalent attachment of **3** to the PSNPs and SiO_2_-NPs could also be kept identical as a prerequisite for the intended nanosensor comparison.

**Figure 4:**
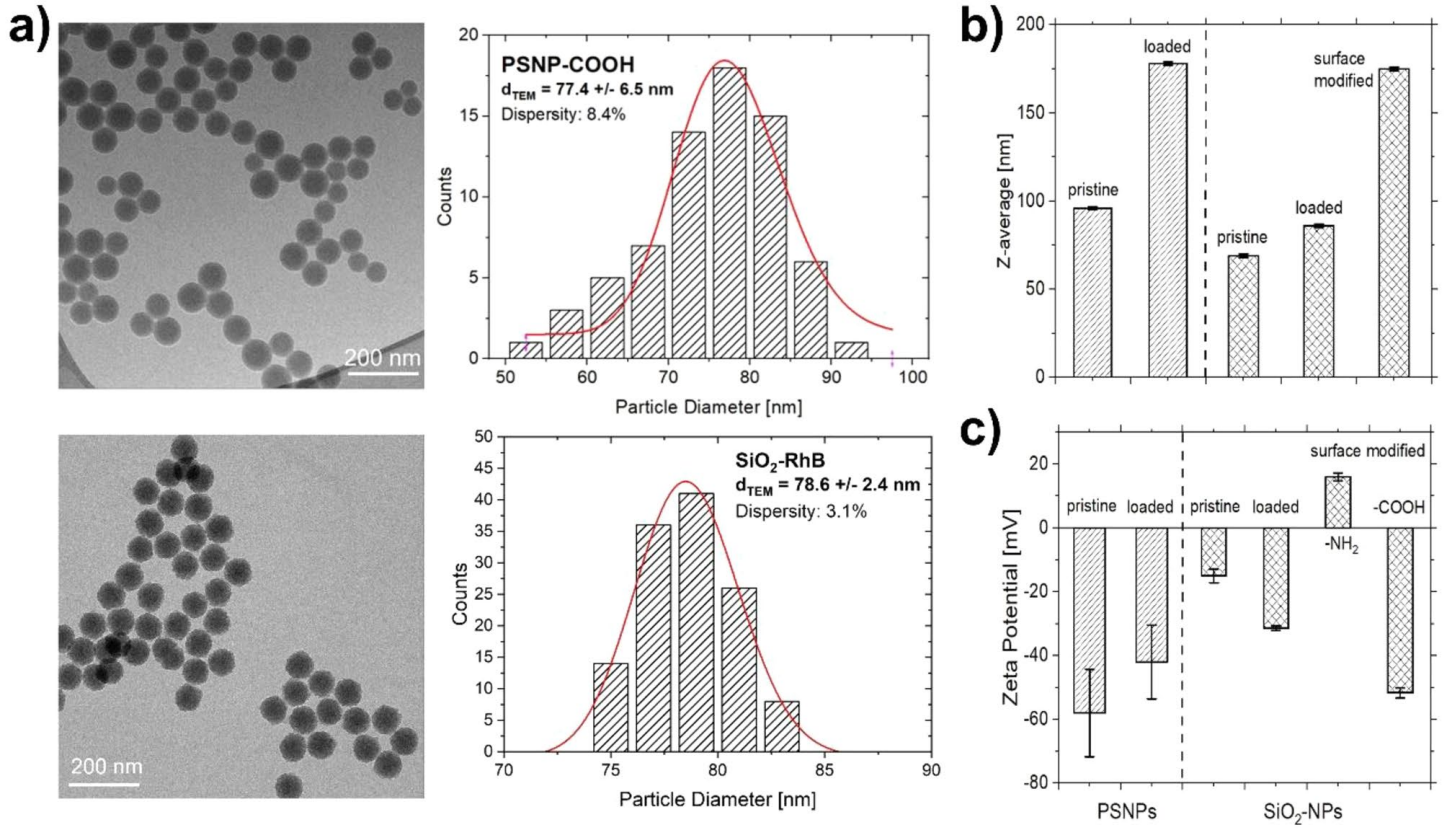
(**a**) TEM images and histograms of the pristine PSNPs and the surface modified SiO_2_-NPs; (**b**) hydrodynamic diameters (z-average) measured by DLS; (**c**) zeta potential measurements after different synthesis steps.

The PSNPs and SiO_2_-NPs were characterized by dynamic light scattering (DLS) and zeta potential measurements before and after the loading with the reference dyes, as well as after the surface modification with carboxylic acid groups (Fig. 4b,c, and SI, Figs. S22 and S23). The observed increase in z-average after dye loading of the PSNPs (177 ± 7 nm) compared to the pristine particles (96 ± 0.2 nm) is attributed to the influence of the swelling procedure. However, the hydrodynamic diameter of the particles, determined from the number distribution, showed a smaller increase in particle size from 68 ± 14 to 74 ± 26 nm. While the z-average of pristine SiO_2_-NPs (69 ± 1 nm) only increased by about 20 nm during the cladding of the dyed silica shell (SiO_2_-RhB), the particle size significantly increased after the two-step surface modification to 164 ± 3 nm and 176 ± 1 nm for SiO_2_-RhB-NH_2_ and SiO_2_-RhB-COOH, respectively. Transmission electron microscopy (TEM) measurements of the PSNPs and SiO_2_-NPs performed to determine the size of the particle cores and the particle morphology gave particle sizes of 77.4 ± 6.5 nm (pristine PSNPs), 60.5 ± 1.6 nm (pristine SiO_2_-NPs, Fig. 4a, and SI, Fig. S21d), and 79.3 ± 2.4 nm (SiO_2_-RhB-COOH, after surface modification with COOH groups; Fig. 4a). The PSNPs exhibited a nearly spherical shape with a smooth particle surface while the SiO_2_-NPs showed a rougher surface due to their synthesis in multiple steps. Zeta potential measurements of the carboxylated PSNPs and SiO_2_-NPs gave negative zeta potentials of − 58 ± 14 mV and − 23.9 ± 0.7 mV, indicative of a high colloidal stability of both particles. The number of reference dye molecules per NP was determined spectroscopically to 130 NR molecules for the PSNPs and to 223 RhB-APTES molecules (APTES: 3-aminopropyl)triethoxysilane) for the SiO_2_-NPs (SI, Figs. S25–S28), after particle dissolution. For the fluorometric quantification, calibration curves of solutions of known concentrations of NR in tetrahydrofuran (THF) and RhB-APTES in aqueous B-R buffer were utilized and the emission spectra of previously dried amounts of PSNP-NR-COOH and SiO_2_-RhB-COOH of known mass dissolved in an aqueous B-R buffer were measured.

Subsequently, the number of accessible COOH groups on the surface of the PSNP-NR-COOH and SiO_2_-RhB-COOH systems was determined by an optical toluidine blue (TBO) assay previously assessed by us for the characterization of the number of carboxylic acid groups on carboxylated polymethylmethacrylate particles^65^. This assay relies on the adsorption of the positively charged dye TBO onto the surface of the negatively charged particles and electrostatic interactions and requires the determination of a stochiometry factor to consider the differencies in size between the dye and the COOH groups. For the comparison of our particle systems, we assumed an identical stochiometry factor for both types of particles. This yielded a functional group density of 35 nmol/mg and 24.5 nmol/mg for the PSNPs and SiO_2_-NPs, respectively. Quantification of the total number of COOH groups on the surface of the carboxylated PSNPs was done by a conductometric titration yielding a total COOH density of 167 ± 5 nmol/mg (SI, Fig. S29). This conductometric method is not suitable for SiO_2_-NPs. Assuming only about 20% of the COOH groups on the particle surface are accessible for the functionalization with a fluorophore such as optical probe **3**, the appropriate amount of **3** was covalently attached to the carboxylated particles by EDC/NHS coupling chemistry, resulting in the green–red emissive pH nanosensors PS-NR-**3** and SiO_2_-RhB-**3**. This is displayed in Fig. 3. The photometric quantification of **3** on the surface of the PSNPs and SiO_2_-NPs (SI, Figs. S16 and S32) revealed a comparable number of surface-bound probe molecules, i.e., 0.4 molecules/nm of **3** for PSNP-NR-**3** (equaling a dye conjugation of 76% of the accessible COOH groups) and 0.4 molecules/nm of **3** for SiO_2_-RhB-**3** (equaling a nearly quantitative functionalization of the accessible COOH groups). The NP surface modification was additionally confirmed by FT-IR spectroscopy (SI, Fig. S31).

### pH-dependent optical properties of the nanosensors

**Figure 5:**
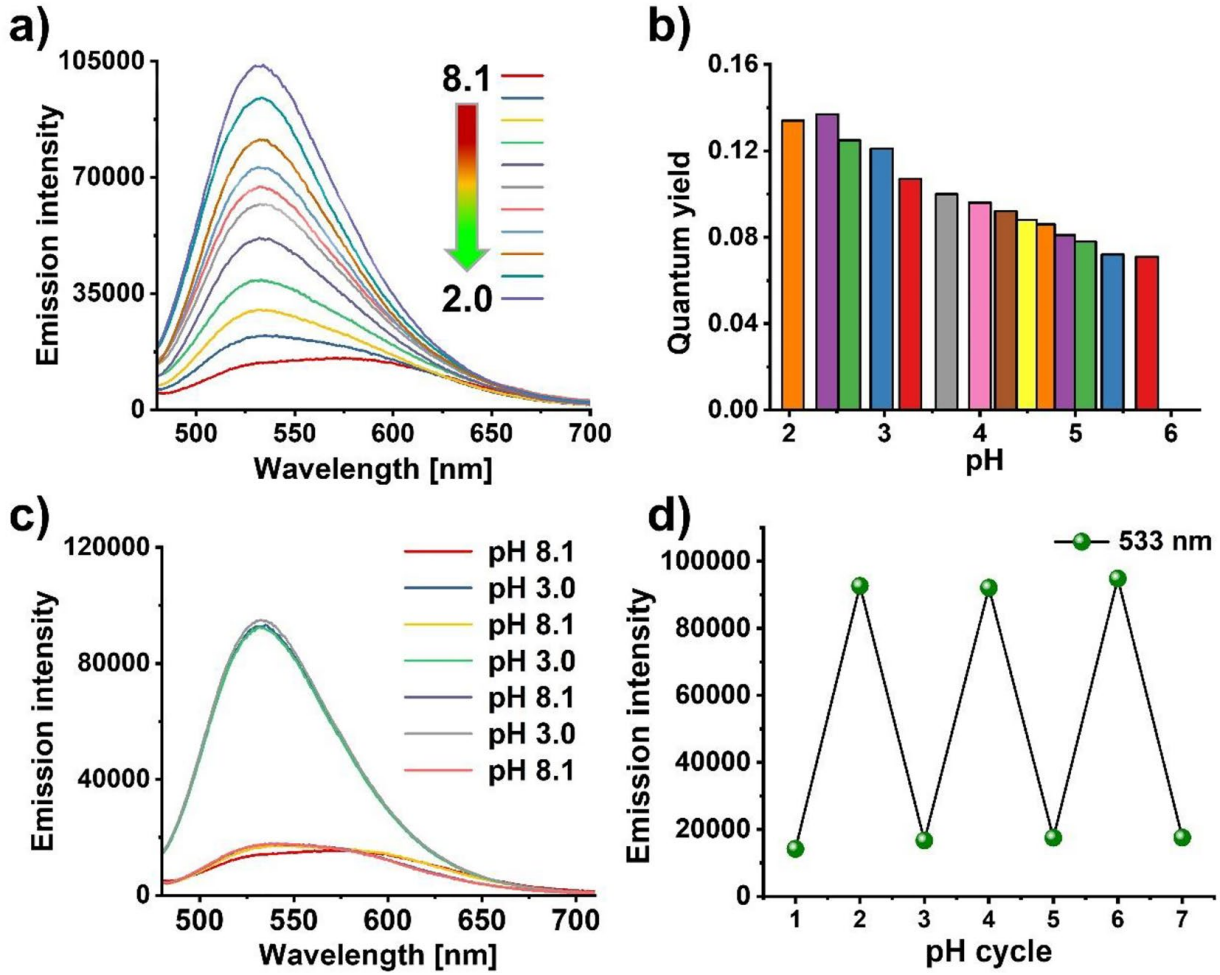
pH-dependent fluorescence of PSNP-NR-**3**. (**a**) Emission spectra at different pH values; (**b**) bar diagram of the pH-dependence of the fluorescence quantum yields in the pH range of 8.1– 2.0; (**c**,**d**) reversibility study of PSNP-NR-**3** involving the measurement of the emission spectra and the fluorescence intensity obtained by varying the pH from 8.1 to 3.0 in cycles in an aqueous B-R buffer (25 mM). Excitation was at *λ*_*Ex*_ of 405 nm.

The pH sensitivity of the nanosensors PSNP-NR-**3** and SiO_2_-RhB-**3** was fluorometrically examined under similar conditions as used for molecular probe **3** (absorption spectra, see SI, Figs. S33 and S34). At pH 8.1, excitation of the nanosensors at λ_Ex_ of 405 nm led to emission bands at 578 nm originating from the reference dyes NR and RhB (Fig. 5a; and SI, Fig. S35). At more acidic pH values, a new fluorescence band appeared at about 530 nm corresponding to the emission of surface bound probe **3** switched ON under these conditions. This green fluorescence became more prominent with decreasing pH. The normalized emission spectra of the nanosensors obtained at different excitation wavelengths of the reference dyes NR (λ_Ex_ = 510 nm), RhB-APTES (λ_Ex_ = 540 nm), and **3** (λ_Ex_ = 405 nm) at pH 4.0 are displayed in the SI in Fig. S36. Measurements of the fluorescence quantum yields of both nanosensors at different pH values between 8.1 and 2.0 revealed maximum fluorescence quantum yields of 14% for both PSNP-NR-**3** and SiO_2_-RhB-**3** (Fig. 5b). This reduction of the fluorescence quantum yield of probe **3** from initially 37% is attributed to surface effects caused by the relatively short distance between the pH-responsive dye and the particle surface and was not further examined. Finally, the reversibility of the pH-sensitivity of PSNP-NR-**3** and SiO_2_-RhB-**3** was assessed by varying the pH between 8.1 and 3.0/4.0 in cycles as well as possible interferences. We deliberately chose a broad pH range for this reversibility study although pH values below 4.2 (lysosomes) are not observed in cells to pave the road for other applications such as corrosion studies. The results shown in Fig. 5c,d, and in the SI (Fig. S35c,d) confirmed the reversibility of the switching ON and OFF of the green naphthalmide fluorescence of both nanosensors.

### Stability studies at different pH and in different environments

**Figure 6:**
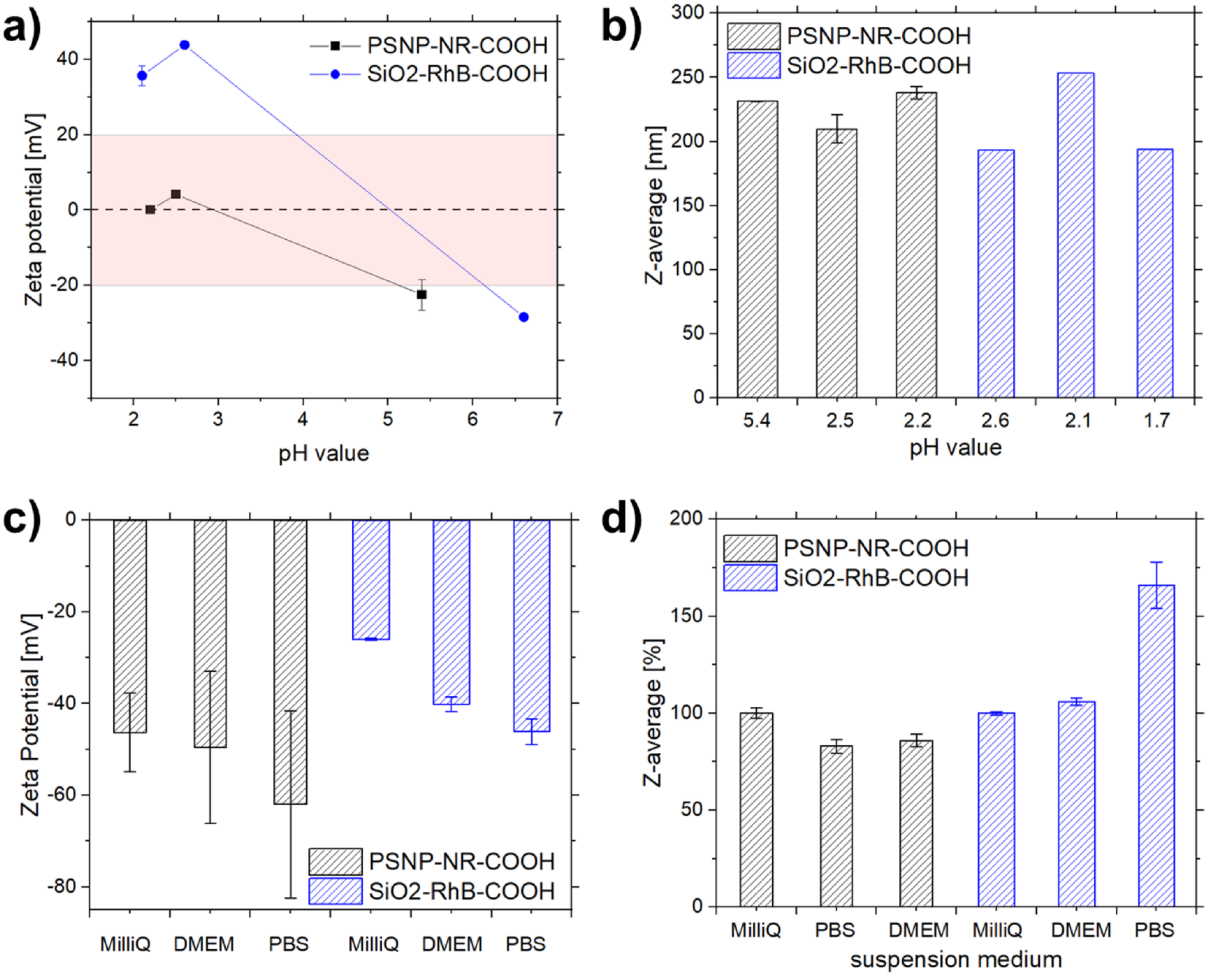
Overview of the results of the stability studies with carboxylated and dye stained PSNPs and SiO_2_-NPs utilizing zeta potential and DLS measurements at various pH values (**a**,**b**) and in different dispersion media (**c**,**d**).

As a prerequisite for the intended cell studies, the colloidal stability of PSNP-NR-**3** and SiO_2_-RhB-**3** and their particle precursors were investigated at pH values between 7.0 and 2.0 in MilliQ water, PBS, and the cell culture medium DMEM, and evaluated by DLS and zeta potential measurements. The zeta potential measurements at different pH values (Fig. 6a) showed a decreased colloidal stability of PSNP-NR-COOH NPs for pH values between pH 2–3, while the SiO_2_-RhB-COOH NPs exhibited a good colloidal stability at all pH values examined. The particle size (Fig. 6b) varied slightly for both types of NPs at the different pH values. The stability studies of both nanosensors at 37 °C in MilliQ water, PBS, and DMEM revealed no significant changes of the particle size and only slight changes of the zeta potential (Fig. 6c,d).

### Intracellular pH imaging with green–red emissive PSNP- and SiO_2_-NPs based nanosensors

**Figure 7:**
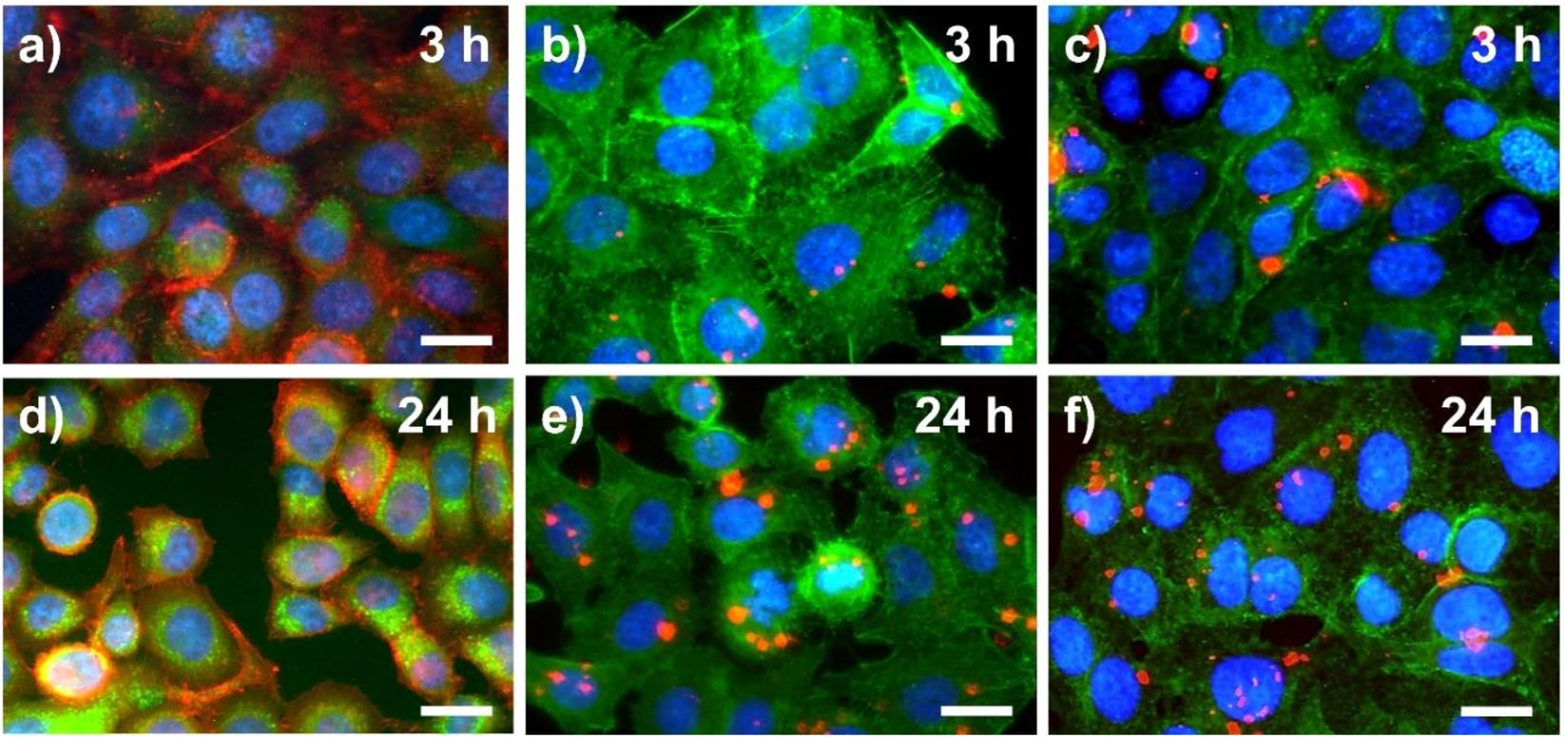
Epifluorescence images of the uptake of optical probe **3** (**a**,**d**), and the pH nanosensors PSNP-NR-**3** (**b**,**e**) and SiO_2_-RhB-**3** (**c**,**f**) by A549 cells measured after different incubation times, 3 h and 24 h, respectively. Prior to the fluorescence microscopy studies, the cells were incubated alive, fixed with 4% paraformaldehyde (PFA), and then co-stained with DAPI (cell nuclei, blue) and partly phalloidin-Alexa 488 (actin filaments, shown in red for (**a**,**d**) and in green for (**b**,**c**,**e**,**f**)) 1:10 dilution of probes dispersed in MilliQ water with PBS buffer. Excitation was carried out with an Osram 50 W/ACL1 Cz HBO Mercury vapor short-arc lamp and the fluorescence was monitored with emission filters set to λ_Em_ = 470 nm (green channel) and to λ_Em_ = 560 nm (red channel). For the detection of the fluorescence of DAPI Leica filter cube A (λ_Em_ = 340/380 nm (blue channel) was used. All images show a scale bar of 10 µm.

To assess the suitability of both pH nanosensors for bioimaging studies, we performed in vitro uptake studies with molecular probe **3** and the pH nanosensors PSNP-NR-**3** and SiO_2_-RhB-**3** using the cell line A549. This cell line presents a model for human alveolar epithelial type II cells. The A549 cells were incubated with 1:10 diluted stock solutions of **3** (containing 37 µg) or 100 µg of each of the two nanosensors for 30 min up to 24 h. Cellular uptake was then examined by epifluorescence measurements (Fig. 7). To visualize the cells and to localize optical probe **3** and both pH nanosensors, the cell nuclei were co-stained with 4’,6-diamidino-2-phenylindole (DAPI, see Fig. 7, blue) and the actin filaments of the cytoskeleton were co-stained with phalloidin-Alexa 488 (Fig. 7a,d-red; b,c,e,f-green). Qualitative image analysis indicated the cellular uptake of **3** after 30 min (SI, Fig. S37) and its subsequent accumulation at longer incubation times (Fig. 7a,d, and SI, Fig. S37). The exposure of the cells to both types of pH nanosensors led to the internalization of both nanomaterials. After 3 h, the cellular uptake of the SiO_2_-RhB-**3** particles was slightly higher than that of the PSNP-NR-**3** particles, yet a similar uptake was observed for both nanosensors after 24 h (Fig. 7b,c,e,f, and SI, Fig. S37 and S38)^66^. The appearance of the green naphthalimide fluorescence indicated cellular uptake and the localization of the naphthalimide-based sensors in an acidic microenvironment, as the green emission of the naphthalmide-based PET probe is only switched ON at acidic pH values below 6. This provides a clear hint for the localization of the probes near or in acidic organelles such as the lysosomes involved in autophagy, protein degradation, apoptosis, and cell defence mechanism^9,10^. Figure 7 and Fig. S38 in the SI also highlight the advantages of dual color emissive nanosensors, that are detectable in the merged blue-green channel and additionally in the red channel, compared to single color fluorescent molecular probe **3**.

**Figure 8:**
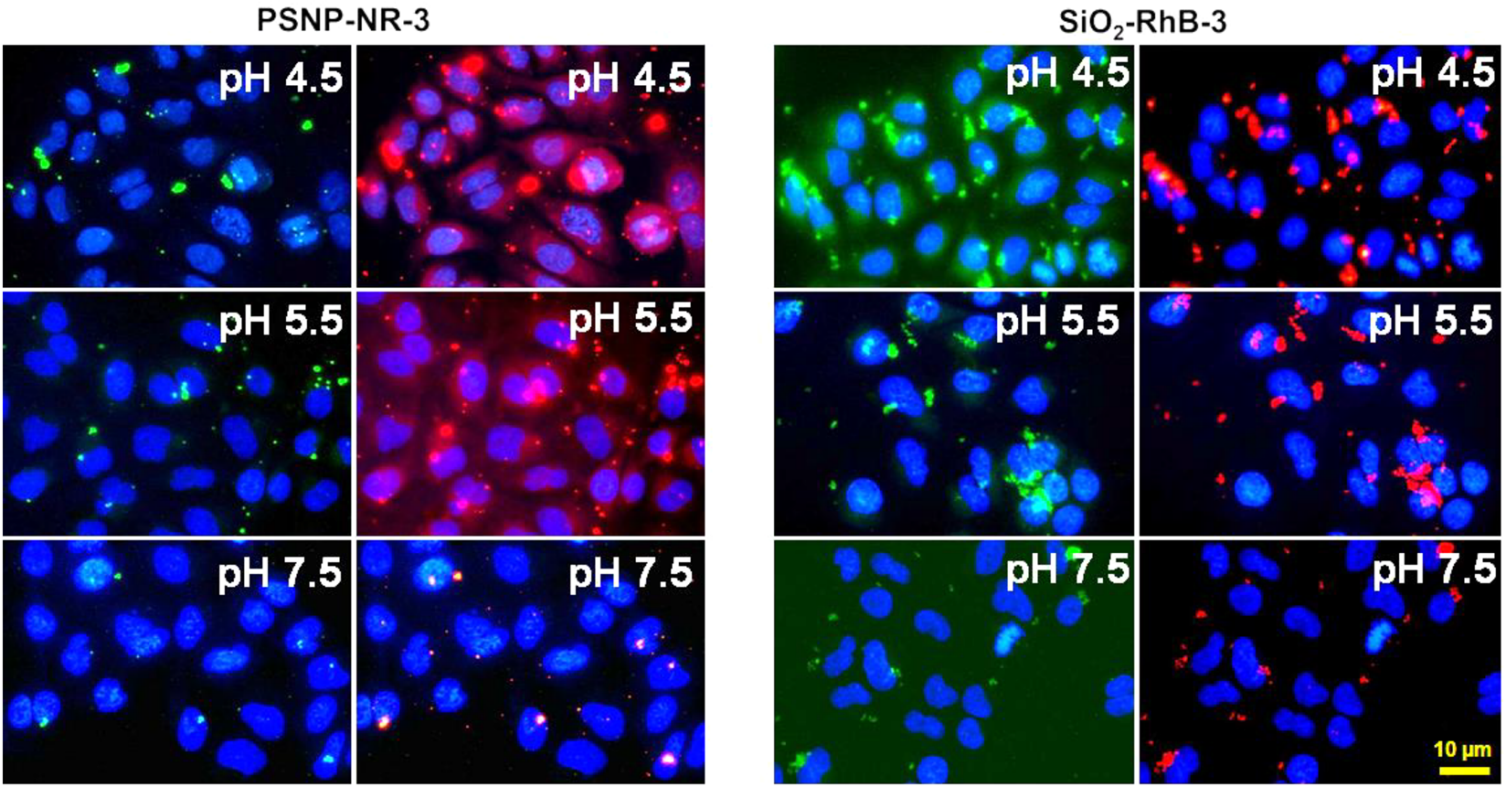
Epifluorescence images of fixed A549 cells incubated with solutions of pH nanosensors PSNP-NR-**3**, and SiO_2_-RhB-**3** in the presence of the ionophore nigericin for 30 min at different pH values; co-staining with DAPI, fixation with PFA. Excitation was carried out with an Osram 50 W/ACL1 Cz HBO Mercury vapor short-arc lamp and the fluorescence was monitored with emission filters set to λ_Em_ = 470 nm (green channel) and to λ_Em_ = 560 nm (red channel). For the detection of the fluorescence of DAPI Leica filter cube A (λ_Em_ = 340/380 nm (blue channel) was used. All images show a scale bar of 10 µm. For all images the autofluorescence of the cells was subtracted by using images of a control measurement done under identical conditions without the addition of particles.

To study the influence of intracellular pH changes on the fluorescence response of the two nanosensors in more detail, the H^+^/K^+^ ionophore nigericin was used to homogenize the intracellular pH and the external pH of the culture media^67,68^, employing a literature protocol^69^. Subsequently, living cells were incubated with optical probe **3** and both nanosensors dispersed in sterile PBS solution of varying pH values (pH 4.5, pH 5.5, and pH 7.5), in the presence of this ionophore. After 30 min the cells were fixed with PFA to maintain the fluorescence signal intensities. While the cells cultivated with a physiological culture medium (pH 7.4) only have an acidic pH value of approximately 4.5 in the lysosomal compartment, the pH value changed during incubation with the ionophore and culture medium, yielding different pH values also in the cytoplasm. To enable a comparison of the intensities of the fluorescence signals measured with the epifluorescence microscope, all images were recorded with the same exposure times. As shown in Fig. 8, the fluorescence signals of both nanosensors increased with decreasing pH values. At pH 7.5, fluorescence signals could be observed only the acidic lysosomal compartment, while at pH 4.5, fluorescence signals also originated from the now acidic cytoplasm. With the instrument settings applied for the epifluorescence microscope measurements, i.e., excitation with a mercury vapor short-arc lamp and a 470 nm (“green”) and a 560 nm (“red”) filter in the emission channel, fluorescence signals could be detected in both the 470 nm and 560 nm channel (Fig. 8). In the case of the PSNP-NR-**3** particles, the fluorescence intensity increase under acidic conditions was more pronounced in the red fluorescence channel, while for the SiO_2_-RhB-**3** particles, the fluorescence enhancement was stronger in the green channel. This dual emission can open variable application possibilities for the use of these green–red emissive nanosensors in biological models, e.g., the detection of acidic environments in tumor tissues. These possibilities can be expanded through the utilization of other filter settings or other measurement conditions in conjunction with a confocal laser scanning microscope (CLSM) enabling laser excitation at defined excitation wavelengths and more choices of emission filter settings and thereby a better spectral discrimination of the two fluorescence signals.

**Figure 9:**
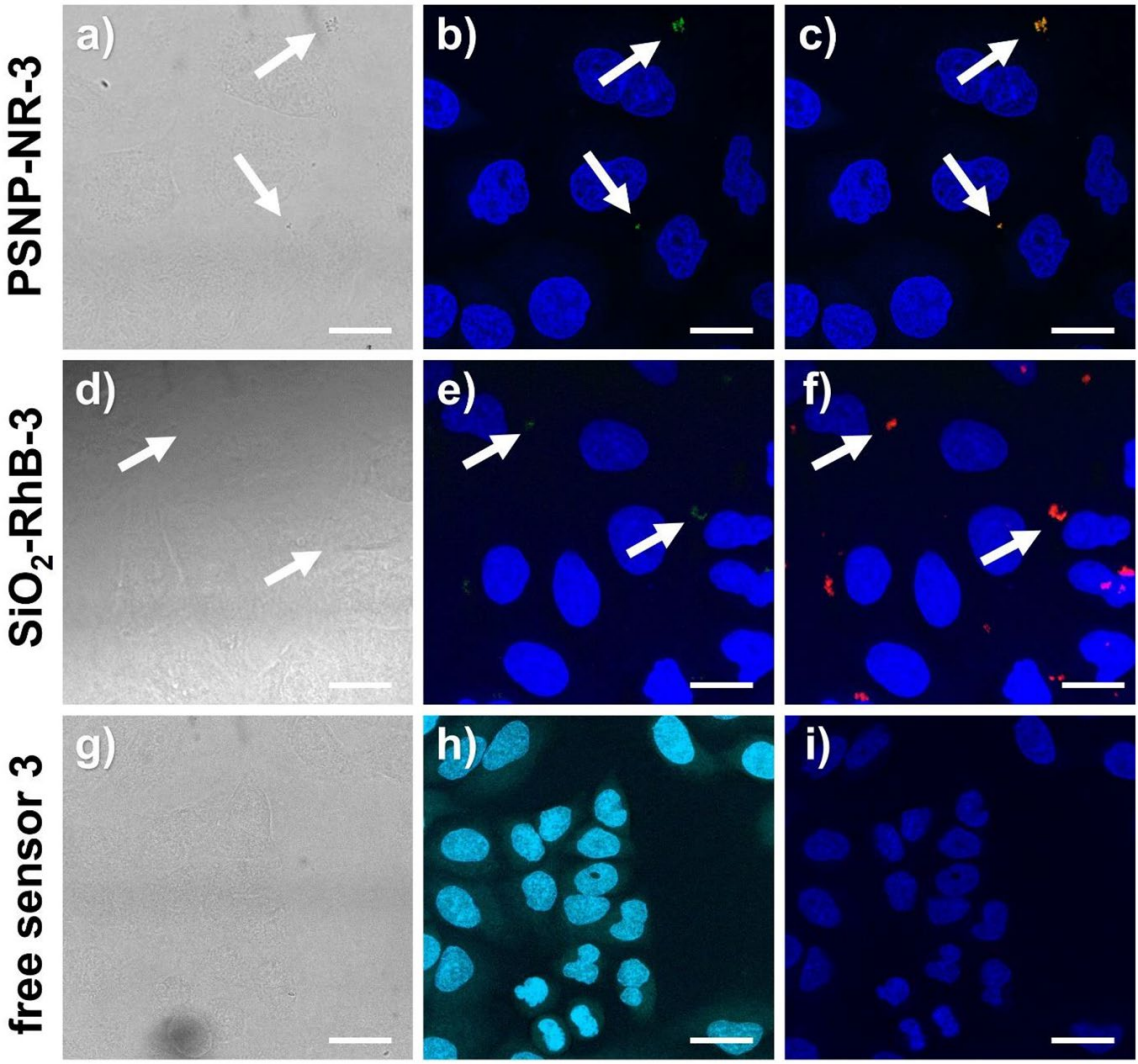
CLSM images of the fixed A549 cells (PFA) that were previously incubated with PSNP-NR-**3** (**a**–**c**, particle concentration 100 µg/mL), SiO_2_-RhB-**3** (**d**–**f**, particle concentration 100 µg/mL), and **3** (**g**–**I**, dye concentration 35 µg/mL) for 30 min at a pH 4.5 in the presence of the H^+^/K^+^ ionophore nigericin. The nuclei were stained with DAPI. Transmitted light (**a**,**d**,**g**); merged blue (DAPI) and green (pH probe **3**) channels: (**b**,**e**,**h**); and merged blue (DAPI), green (pH probe **3**) and red (reference dyes, NR or RhB) channels: (**c**,**f**,**i**). The following measurements conditions were used: λ_Ex_ = 405 nm, λ_Em1_ = 420-480 nm and λ_Em2_ = 520–560 nm for DAPI and **3**, and λ_Ex_ = 560 nm and λ_Em_ = 570-660 nm for the red reference dyes. All images show a scale bar of 10 µm.

Subsequently, we performed first CLSM studies with fixed A549 cells incubated with the optical probe **3** and both nanosensors at pH values of 4.5 and pH 7.5 to confirm the epifluorescence imaging results (Fig. 9 and SI, Fig. S39). These measurements enable a better morphological assignment. In addition, the more specific choice of the excitation wavelength and the emission filter settings allows a more selective recording of the green and red fluorescence. The disadvantage is, however, the lower sensitivity and the increased risk of bleaching effects. Depending on their location within the cells, the nanosensor emission could be detected in the red and green channel of the CLSM, while **3** was only visible in the green channel at pH 4.5 and not fluorometrically detectable at pH 7.5 (SI, Fig. S39h), as to be expected. To support the internalization of the NPs by the cells, a z-stack was measured (SI, Fig. S40) which confirmed the cellular uptake of the two nanosensors. The fluorescence detected in the green channel in the absence of nigericin at pH 7.5 in the medium (SI, Fig. S40) indicates that some nanosensor particles already entered acidic cell compartments, most likely the lysosomes. For a better co-localization assignment of the endosomes and lysosomes, however, live cell imaging studies are needed that were beyond the scope of this comparative screening study.

## Conclusion and outlook

We developed a set of dual color emissive ratiometric pH nanosensors utilizing polystyrene (PS) and silica (SiO_2_) nanoparticles (NPs) core stained with a red emissive pH-inert reference dye with an always ON fluorescence and surface functionalized with a green-fluorescent pH-responsive naphthalimide probe bearing a piperazine and a morpholine unit favoring lysosomal targeting. The ICT emission of this water-soluble pH probe, designed for lysosomal targeting, is quenched by PET from the unprotonated nitrogen atoms of the piperazine and morpholine moieties at basic and neutral pH values and switched ON at pH values < 6. This was confirmed by pH titrations monitored optically and by NMR spectroscopy. Aiming for a comparative screening of the performance of inorganic and organic matrices typically used for nanosensors such as PS and SiO_2_, the nanosensor design and preparation were performed to provide closely matching physico-chemical and optical properties relevant for life sciences applications. This included the particle size, choice of the sensor dye, spectroscopic properties of the reference dyes, nanosensor surface chemistry, and number of surface-bound sensor dyes as supported by the subsequent nanosensor characterization, as well as the nanosensor concentration used for all characterization and cell uptake studies.

A comparison of the cellular uptake and sensing potential of both nanosensors using A549 cancer cells and epifluorescence and confocal fluorescence microscopy confirmed the cellular uptake of both types of nanosensors. Dual color emission was observed under acidic conditions required to switch ON the green fluorescence of the naphthalimide pH probe and suggested nanosensor penetration into acidic cell compartments. This underlines the suitability of both nanosensors for the fluorescence imaging of intracellular pH and lysosomal tracking. Cellular uptake of the SiO_2_-based nanosensor particles was slightly more efficient than that of the PS-based nanosensor particles. The silica nanosensors also revealed an improved stability. This provides clear evidence of the importance of the carrier matrix for nanosensor performance. Our results also underline the relevance of the choice of filter settings for the optimum read out of dual emissive probes and nanosensors.

In the future, we plan to further perform similar studies with analogously designed PS and SiO_2_ nanosensors of different size to gain a better insight in the influence of the nanosensor matrix on nanosensor performance in cellular imaging studies. In addition, we will also assess different dye combinations such as the combination of our green emissive naphthalimide PET probe with a reference dye revealing a longer-wavelength emission than Nile Red or rhodamine B or the substitution of our neutral naphthalimide probe for a pH-responsive zwitterionic Bodipy dye with a green LE-type PET-operated fluorescence.

## Materials and methods

### Materials

All chemicals used for the particle syntheses were obtained in the highest purity available, while all solvents used for the optical measurements were of spectroscopic grade. The chemicals, reagents, and solvents were employed as received, unless otherwise stated. For all syntheses and purification steps, ultrapure water was used (18.2 MΩ, MilliQ water). Tetraethoxysilane (TEOS, 99%), acrylic acid (AA, 99%), rhodamine B isothiocyanate (≥ 95%), anhydrous sodium hydroxide (≥ 98%), anhydrous potassium carbonate (≥ 99%) and *N*,*N*’-dimethylformamide (DMF, anhydrous, 99.8%) were purchased from Sigma Aldrich. 4-(2-aminoethyl) morpholine, triethylamine (NEt_3_), toluene (spectr. grade), styrene (≥ 99.0%), succinic anhydride (98%) and potassium persulfate (PPS, ≥ 99.0%, p.a.) were obtained from Merck KGaA. Ethanol (abs., 99.9%), cyclohexane (99.5%), dichloromethane (DCM), methanol (MeOH), DMF, ethyl acetate (EtOAc), acetonitrile (MeCN), hexane, glacial acetic acid and tetrahydrofuran (THF, 99.9%, p.a.) were obtained from Labsolute, and pyridine, Na_2_CO_3_ were acquired from Chemsolute. 1-(2-aminoethyl)piperazine and phosphoric acid (85%) were purchased from Alfa Aesar, while 1-ethyl-3-(3-dimethylaminopropyl)carbodiimide (EDC) and sodium dodecyl sulfate (SDS, ≥ 97%, p.a.) were purchased from Carl Roth GmbH + Co. KG. 4-bromo, 1–8 naphthalic anhydride, N-hydroxy-sulfosuccinimide sodium salt (sulfo-NHS), 3-aminopropyl)triethoxysilane (APTES) were purchased from abcr. Nile Red was purchased from Fluka Analytical.

### Synthesis of the water-soluble pH probe 3

#### Step 1: Synthesis of 4-bromo-N-4-(2-Aminoethyl) morpholine-1–8 naphthalimide (2)

4-bromo, 1–8 naphthalic anhydride (277 mg, 1 mmol) and 4-(2-Aminoethyl) morpholine (131 µL, 1 mmol) were taken in EtOH and heated at 50° C for 5 h. After complete reaction monitored by thin layer chromatography (tlc) the reaction mixture was concentrated and added to ice-cold water. The obtained precipitate was filtered and washed with 10% Na_2_CO_3_ solution and dried. The pure product was obtained by column chromatography using ethyl acetate and hexane as eluents as an off- white shining powder. 311 mg; Yield: 80%. ^1^H NMR (CDCl_3_; 500 MHz): δ (ppm) 8.64–8.62 (dd, 1H), 8.56–8.54 (dd, 2H), 8.39–8.38 (d, 1H), 8.03–8.02 (d, 1H), 7.85–7.82 (m, 1H), 4.36–4.34 (m, 2H), 3.71 (b, 4H), 2.67–2.66 (b, 6H); ^13^C NMR (125 MHz, CDCl_3_) δ (ppm) 163.7, 133.4, 132.1, 131.3, 131.2, 130.7, 130.4, 129.1, 128.1, 123.1, 122.2, 66.7, 56.0, 53.7, 37.07; ESI–MS m/z calculated for C_18_H_17_BrN_2_O_3_; [M + H]^+^ 389.2490, found 389.0541.

#### Step 2: Synthesis of Lysosomal targeting water soluble pH sensor; 4-Amino (2-aminoethyl)piperazine-N-4-(2-aminoethyl) morpholine-1,8 naphthalimide (3)

**3** (194 mg, 0.5 mmol), 1-(2-aminoethyl)piperazine (78.7 µL, 0.6 mmol) and a few drops of triethylamine (NEt_3_) were dissolved in pyridine. The reaction mixture was stirred under reflux overnight. After completed reaction as monitored by thin layer chromatography (TLC), the reaction mixture was concentrated and purified by column chromatography with dichloromethane and methanol mixture to obtain a dark yellow solid. ^1^H NMR (CD_3_OD; 500 MHz): δ (ppm) 8.45–8.42 (m, 2H), 8.39–8.37 (m, 1H), 7.73 (m, 1H), 7.30–7.29 (m, 2H), 4.29–4.27 (m, 2H), 3.70 (m, 4H), 3.36 (m, 4H), 3.20 (m, 2H), 2.91–2.84 (m, 6H), 2.70–2.69 (m, 2H), 2.63 (m, 4H); ^13^C NMR (125 MHz, CD_3_OD) δ (ppm) 168.3, 167.9, 160.1, 136.3, 136.2, 134.2, 134.6, 133.5, 129.7, 129.4, 126.5, 119.7, 118.7, 70.3, 59.8, 58.6, 57.5, 56.7, 40.4, 40.2; ESI–MS m/z calculated for C_24_H_31_N_5_O_3_; [M + H]^+^ 438.2460, found 438.2554.

### Synthesis of the PS and SiO_2_ particles and surface modifications

#### Synthesis of Nile Red loaded and carboxy functionalized PSNP (PSNP-NR)

The spherical, carboxy functionalized PSNPs (PSNP-COOH) were synthesized by an emulsion polymerization under argon atmosphere and loaded with Nile Red (NR) according to a procedure adapted from Nirmalanthan-Budau et al.^55^ For the synthesis, 400 µL of an aqueous solution of the radical initiator PPS (0.148 mM) was added to a mixture of 5.2 mL of an aqueous solution of the surfactant SDS (0.042 mM) and 1.3 mL styrene monomer at 70 °C. After 1 h of stirring, 30 µL AA in 470 µL of water were added dropwise. The mixture was kept at 70 °C and stirred for three more hours before cooled to r.t.. The resulting particle solution was diluted fivefold and centrifuged two times for 2 min at 13,500 rcf, the supernatants were collected and combined to create a stock solution with a particle concentration of 28 mg/mL (determined by weighing).

For PSNP swelling and dye staining, 100 µL of NR in THF (2 mM) were quickly added to 3 mg of PSNP-COOH stock particles in 600 µL of MilliQ water and placed in a plate shaker at 300 rpm and r.t. for 30 min. 300 µL of MilliQ water were added, and the NPs were redispersed and centrifuged two times for 40 min at 16,000 rcf followed by discarding the supernatants. The PSNP dispersions were combined and centrifuged again to yield a dispersion of NR-stained PSNPs in MilliQ water with a particle concentration of 10 mg/mL.

#### Synthesis of RhB-APTES loaded SiO_2_-NP (SiO_2_-RhB)

SiO_2_-NPs were synthesized as described in the literature, using a l-arginine controlled hydrolysis of TEOS in a biphasic water/cyclohexane system^63,64^. 91 mg (0.522 mmol) of l-arginine were dissolved in 69 mL of MilliQ water and 4.5 mL of cyclohexane was added. After heating the biphasic water / cyclohexane system to 60 °C, 5.5 mL (0.025 mmol) of TEOS were added to the upper layer and the reaction mixture was stirred for an additional 20 h at 150 rpm. To purify the obtained SiO_2_ seeds with an average size of 25 nm, a dialysis step against water (4 L, water exchange after 30 min, 1 h, 2 h, and 4 h) with a dialysis membrane (Nadir, Carl Roth GmbH, molecular weight cut-off: 10–20 kDa) was performed. Subsequently, the obtained SiO_2_ seeds were used to grow larger SiO_2_-NPs. 10 mL of the SiO_2_ seeds were mixed with 36 mL of MilliQ water and 14 mg (0.08 mmol) of l-arginine, before 5 mL of cyclohexane was added. After heating to 60 °C, 3.52 mL (0.016 mmol) of TEOS were added and the reaction mixture was stirred for 20 h at 150 rpm. Purification of the SiO_2_-NPs was performed as described for the SiO_2_ seeds. To obtain 80 nm large SiO_2_-NPs loaded with RhB-APTES, the regrowth step was performed three times. In the last regrowth step, 0.05 mL (0.9 µmol) of RhB-APTES in ethanol was injected into the aqueous phase 20 min after the addition of TEOS.

#### Surface modification of SiO_2_-RhB (SiO_2_-RhB-COOH)

The particle surface of the SiO_2_-RhB was modified by a two-step post-synthetic reaction. In the first step, amino groups were grafted onto the particle surface using APTES. Therefore 10 mg (20.56 nmol/L of particles) of SiO_2_-RhB were diluted in 10 mL of ethanol and stirred at r.t. under a continuous argon flow. Next, 13.6 µL (0.058 mmol) of APTES was added under constant stirring and the reaction mixture was allowed to stir at 400 rpm for another 20 h. To purify the obtained aminated SiO_2_-NPs, they were centrifuged at 15,000 rcf and washed three times with ethanol. After the last washing step, the particles were redispersed in 5 mL of anhydrous DMF. In the second surface modification step, succinic anhydride reacted with the amino groups on the ligand periphery of the aminated SiO_2_-NPs. Therefore, succinic anhydride (1.25 equiv. of the mol of APTES used in the amination process) was added dropwise to the particle suspension in DMF at 45 °C and stirred at 400 rpm overnight under an Ar atmosphere. Purification of the particles was performed by centrifugation at 15,000 rcf and washing with MilliQ water thrice. Finally, the particles were redispersed in 5 mL of MilliQ water.

#### Labeling of SiO_2_-RhB-COOH and PS-NR-COOH with probe 3

pH probe **3** was covalently attached to the surface of the silica and polystyrene particles using the same reaction conditions. 3 mg each of SiO_2_-RhB-COOH and PS-NR-COOH were taken with *N*-hydroxy sulfo succinimide (s-NHS, 5 mg, 0.023 mmol) and *N*-(3-Dimethylaminopropyl)-*N*-ethylcorbodiimide hydrochloride (EDC, 4.5 mg, 0.023 mmol) in 500 µL of MilliQ water and stirred at r.t. for 1 h. pH probe **3** was dissolved in 100 µL of MilliQ water and then added to the particle suspension. Then, 400 µL of PBS buffer (pH 8) were added to the reaction mixture and stirred overnight at r.t. The resulting PSNP-NR-**3** and SiO_2_-RhB-**3** nanosensors were centrifuged for 10 min at 20,000 g and washed one time with PBS buffer and three times with MilliQ water and finally redispersed in 3 mL of MilliQ water.

### Particle characterization

#### Dynamic light scattering (DLS) and zeta potential measurements

DLS and zeta potential measurements of the PSNPs and SiO_2_-NPs with and without dyes and sensors were carried out with a Zetasizer Nano ZS from Malvern Panalytical Ltd. at T = 25 °C in disposable folded capillary cells (DTS1070), also from Malvern Panalytical Ltd. or disposable cuevettes (Sarstedt). All particles were dispersed in MilliQ water for these measurements. Three independent measurements including several runs were performed for each sample during the DLS measurement (back scattering angle 173°, total time 10 min; only one measurement with three runs for the PSNP-COOH and PSNP-NR-COOH particles as well as the PSNP-NR-**3** sensor particles directly after the synthesis)) and zeta potential (total time 5 min, five measurements for PSNPs) measurements. The zeta potential was calculated from the nanoparticle electrophoretic mobility using the Einstein-Smoluchowski theory, while for DLS measurements the hydrodynamic diameter based on the z-average and number distribution was used. A refractive index of 1.4649 for SiO_2_ and 1.4600 for PS was used, respectively.

#### Transmission electron microscopy (TEM)

The particle shape, average particle diameter, and agglomeration state were determined using a Tecnai G2 20 S-Twin from FEI. The particles were ultrasonicated for the measurements, diluted and added onto a TEM grid. The grids were dried overnight, measured, and the particle size distribution was determined representatively for a randomly chosen sample of 50–150 particles using the X-ImageJ software (Version: 1.52 e, winPenPack X-ImageJ Launcher from the National Institute of Health (http://rsb.info.nih.gov/ij/)***.***

#### Conductometric titration

The total amount of (de)protonable COOH groups on the surface of the PSNPs was determined by a conductometric titration with a Modul 856 conductometer from Metrohm at r.t., following a slightly modified procedure previously described^19^. Samples containing at least 20 mg of PSNP-COOH in 80 mL of MilliQ water were titrated with 0.01 M NaOH in 20 µL/5 s steps under argon atmosphere until a final conductivity of 0.12 mS/cm was reached. Prior to the titration of the particle dispersion, the conductivity was adjusted to 0.1 mS/cm with HCl (0.01 M) and NaBr (30 mM).

#### Dye leaking studies

To determine possible dye leakage from the nanosensor core, leaking studies in different application-relevant microenvironments were performed. For this, 150 µL of the particle suspension was filtered through filter units (Ultracel, MWCO: 30 KDa, Merck) and centrifuged for 10 minutes at 12,000 rcf. After centrifugation, the filtrate and particles were diluted in 3 mL of MilliQ water and the emission intensity was measured.

#### Absorption and emission studies

The absorption spectra of the **3** and particles (PSNPs and SiO_2_-NPs) were measured with a Specord 21 spectrometer from Analytik Jena using quartz cuvettes from Hellma. Fluorescence measurements were performed with a calibrated FluoroMax-4 Spectrofluorometer, HORIBA Jobin Yvon with excitation and emission slit widths of 5 nm. The fluorescence quantum yields were absolutely measured with a calibrated stand-alone Quantaurus Hamamatsu integrating sphere setup using an excitation wavelength λ_Ex_ of 405 nm. Photostability studies of the nanosensors at pH 4.0 were done with the spectrofluorometer FSP920 from Edinburgh Instruments equipped with a xenon lamp and λ_Ex_ = 405 nm, with monochromator slit widths set to 4 nm and 6 nm in excitation and emission, respectively.

For the spectroscopic studies, the dye solutions were prepared from a 1 mM stock solution in DMF. For the experiments in aqueous environments, then a solution of the dye (0.1 mM) in MilliQ water was prepared by dilution of this stock solution. 4 µL of this aqueous solution were used for the fluorescence measurement of **3** and 7.5 µL of the 1 mM stock solution for the absorption measurements.

The different pH solutions were made in B-R buffer using acidic solutions containing phosphoric acid, boric acid, and acetic acid and a basic solution of sodium hydroxide. Solutions of different pH were obtained by the mixing of different acid and base solutions. All pH solutions were prepared using MilliQ water. Reversibility experiments were performed by the addition of HCl and NaOH, subsequently to adjust pH values of 4.8 and 8.1 for pH sensor, 4.0 and 8.1 for SiO_2_-RhB-**3** and 3.0 and 8.1 for PSNP-NR-**3** nanoparticles.

The dose/response Eq. (1) was used to calculate the pKa values of the free pH sensor **3** and covalently liked on the surface of silica and polystyrene nanoparticles by sigmoidal curve fitting of the emission intensities with respect to different pH values.

1 $$\mathbf{y}=\mathbf{I}1+\frac{\mathbf{I}2-\mathbf{I}1}{1+{10}^{\left(\mathbf{L}\mathbf{O}\mathbf{G}{\mathbf{x}}_{0}-\mathbf{x}\right)\mathbf{p}}}$$

All aqueous solutions were prepared with MilliQ water (0.055 μS m^−1^; Merck Milli-Q® IQ 700 device). ^1^H NMR and ^13^C NMR spectra of the final sensor dye **3** preparation steps were measured on a Bruker AVANCE III 500 instrument employing CDCl_3_ and CD_3_OD (Deutero GmbH) as solvents and tetramethylsilane (TMS) as an internal reference. The ^1^H NMR titration was performed in D_2_O using a Varian VNMRS500 type NMR spectrometer operating at 499.9 MHz equipped with a Varian OneNMR probe. Optical probe **3** was also measured in DMSO-d_6_ solution followed by exchange by D_2_O to determine the exact structure of the sensor. An Agilent 6210 ESI-TOF mass spectrometer from Agilent Technologies, Santa Clara, CA, USA was used to obtain the mass spectra. All pH measurements of dye **3** and the PSNPs and SiO_2_-NPs were performed with a Mettler Toledo pH-meter Seven Compact Advanced, Gießen, Germany and the calibrated pH electrode Mettler Toledo InLab® Micro. The pH-meter was calibrated with standard buffers of pH 10.00, 7.01, 4.01, and 2.00.

### Cell culture

The human lung cancer cell line A549 were routinely propagated as follows: DMEM medium, with 10% fetal calf serum (FCS), 2% glutamine, and penicillin/streptomycin (all from PAN Biotech) added. Cells were seeded into medium at a concentration of 1 × 10^5^ cells/mL, cultured at 37 °C with 5% CO_2_, and split twice in a ratio of 1:5 per week. For cytochemistry, the cells were seeded at a concentration of 5 × 10^5^ cells/mL in a 24-well culture plate on glass coverslips (Sigma Aldrich), and cultured for 48 h at 37 °C. Thereafter, cells were incubated with normal culture medium or medium containing test substances as optical probes and nanosensors for different times at 37 °C. Afterwards, the cells were fixed with 4% PFA, rinsed and 4,6-diamidino-2-phenylindole (DAPI, Abcam) was used for nuclear counterstaining.

### Microscopy studies

#### Epifluorescence microscopy

Image acquisition of live cells was performed with a Leica DMRB microscope (Leica, Wetzlar, Germany). The images were taken with a digital camera (Spot 32, Diagnostic Instruments) with the same exposure time for all images. Excitation was carried out with an Osram 50 W/ACL1 Cz HBO Mercury vapor short-arc lamp and commercially available filter settings from Leica were used for the detection of the fluorescence of DAPI (blue channel: Leica Filter Cube A ((λ_Emx_ = 340/380 nm) and the green and red emission of the pH-responsive and pH-inert fluorophores (green channel: λ_Em_ = 470 nm; red channel; λ_Em_ = 560 nm).

#### CLSM

Confocal laser scanning microscopy (CLSM) imaging was done on a confocal laser scanning microscope Leica SP8 equipped with a white light laser (Superk Extreme EXW-9 NIM, NKT Photonics, Denmark) and a 405 nm laser diode (LASOS, VLK 0550 T01) using a 100 × oil immersion objective with a numerical aperture of 1.4 (HC PC APO CS2 100x/1.40 OIL). The following sequential measurement conditions were used: λ_Ex_ = 405 nm, λ_Em1_ = 420—480 nm and λ_Em2_ = 520–560 nm for DAPI and **3**, and λ_Em_ = 560 nm and λ_Em_ = 570–660 nm for the red reference dyes. The images and z-scans (step size = 0.3 micrometer) were deconvoluted with the software Huygens Essential (Version 17.04, Scientific Volume Imaging B.V., The Netherlands) with default settings and a maximum intensity projection of the deconvoluted z-stacks was created.

## Supplementary Information

Supplementary Information.

## Data Availability

All data generated or analyzed during this study are included in this published article (and its Supplementary Information files) or are available upon request from the corresponding author.
