# Supplementary material for: Dual color pH probes made from silica and polystyrene nanoparticles and their performance in cell studies
Source: Sci Rep. 2023 Jan 24;13:1321. doi: 10.1038/s41598-023-28203-0 (PMC9873940; doi:10.1038/s41598-023-28203-0)
Supplement: Supplementary file 1 — Supplementary Information. [file 41598_2023_28203_MOESM1_ESM.pdf]

Dual color pH probes made from silica and polystyrene nanoparticles and their performance in cell studies

Priyanka Srivastava[a,‡], Isabella Tavernaro[a,‡], Lena Scholtz[a,b], Claudia Genger[c,d], Pia Welker[c,d], Frank Schreiber[e], Klas Meyer[f] and Ute Resch-Genger[a*]

[a]Division *Biophotonics*, Federal Institute for Materials Research and Testing (BAM), Richard-Willstaetter-Str. 11, 12489 Berlin, Germany.

[b]Institut für Chemie und Biochemie, Freie Universität Berlin, Takustr. 3, 14195 Berlin, Germany.

[c]nanoPET Pharma GmbH, Robert-Koch-Platz 4, 10115 Berlin, Germany.

[d]Charité-Universitätsmedizin Berlin, Charitéplatz 1, 10117 Berlin, Germany.

[e]Division *Biodeterioration and Reference Organisms*, Federal Institute for Materials Research and Testing (BAM), Unter den Eichen 87, 12205 Berlin, Germany.

[f]Division *Process Analytical Technology*, Federal Institute for Materials Research and Testing (BAM), Richard-Willstaetter-Str. 11, 12489 Berlin, Germany.

‡ The authors contributed equally.

*ute.resch@bam.de, Phone: +49 (0)30 8104 1134

ORCID-IDs

Lena Scholtz: 0000-0002-6869-3654

Ute Resch-Genger: 0000-0002-0944-1115

Klas Meyer 0000-0003-1837-3801

1. Characterization of pH probe 3 2

2. Optical spectroscopy of pH probe 3 8

3. Characterization of PSNPs and SiO_2-NPs 11

3.1 Synthesis of reference dyes: Silane derivatives of Nile Red (NR-Silane) and rhodamine B (RhB-APTES) and their embedding into the SiO_2-NPs 12

3.2 Determination of the amount of reference dyes in the particle cores 15

3.3 Determination of the number of total and accessible COOH groups on the particle surface of PSNP-NR-COOH and SiO_2-RhB-COOH 18

3.4 Surface modification of carboxylated PSNPs and SiO_2-NPs with 3 19

4. Optical spectroscopy of nanosensors 20

5. Fluorescence cell microscopy 24

1. Characterization of pH probe 3

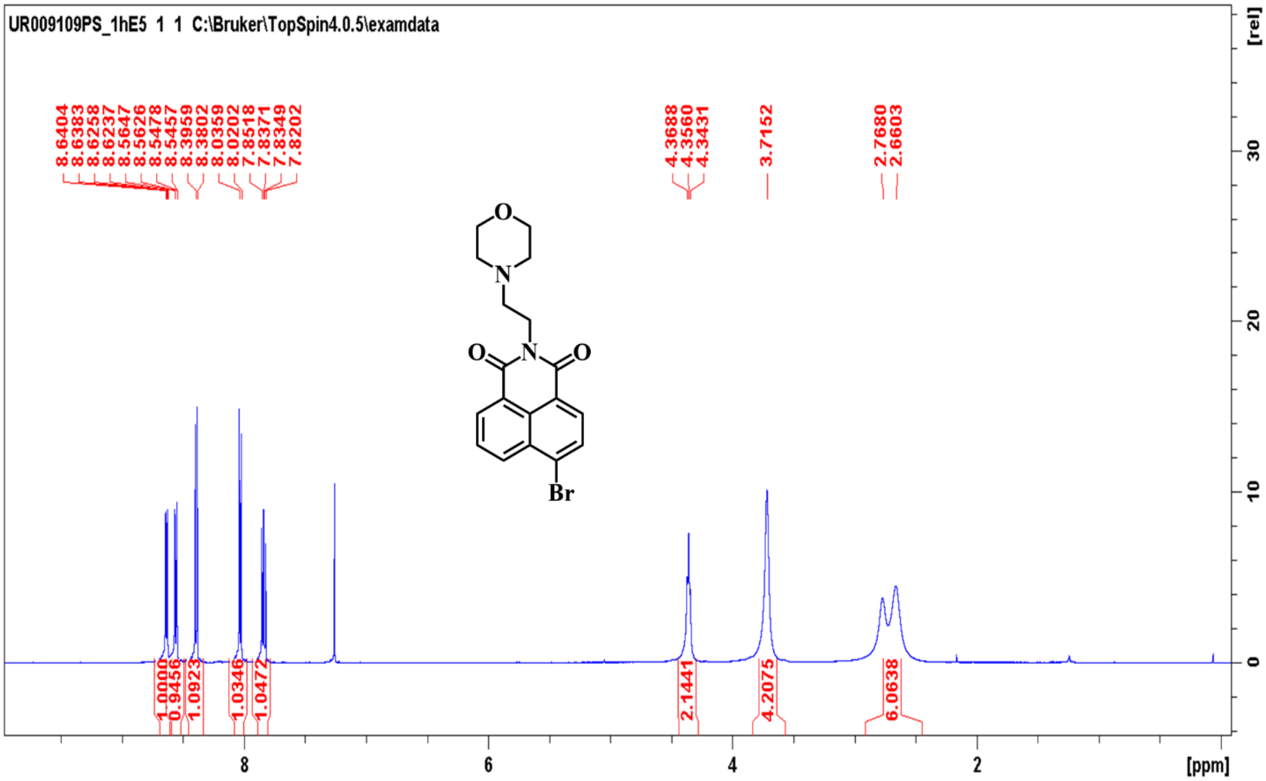

Figure S 1. ^{1}H NMR spectra of compound **2** in $CDCl_3$**.**

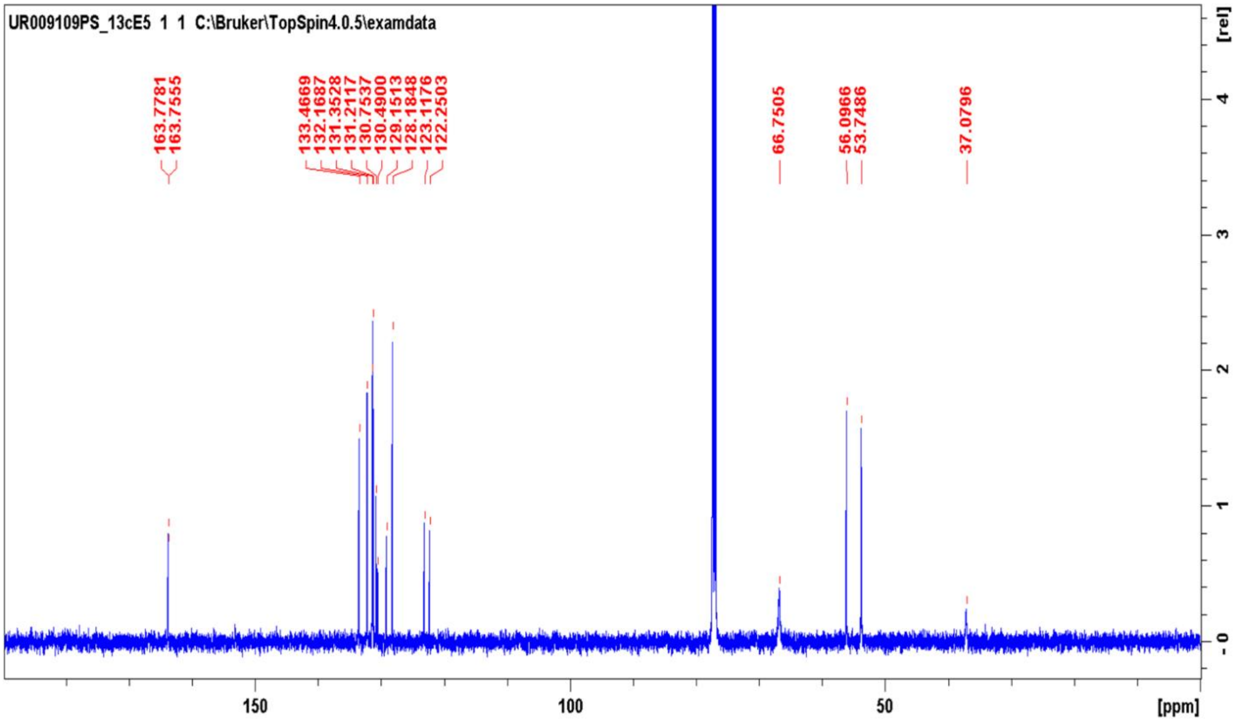

Figure S 2. ^{13}C NMR spectra of compound **2** in $CDCl_3$**.**

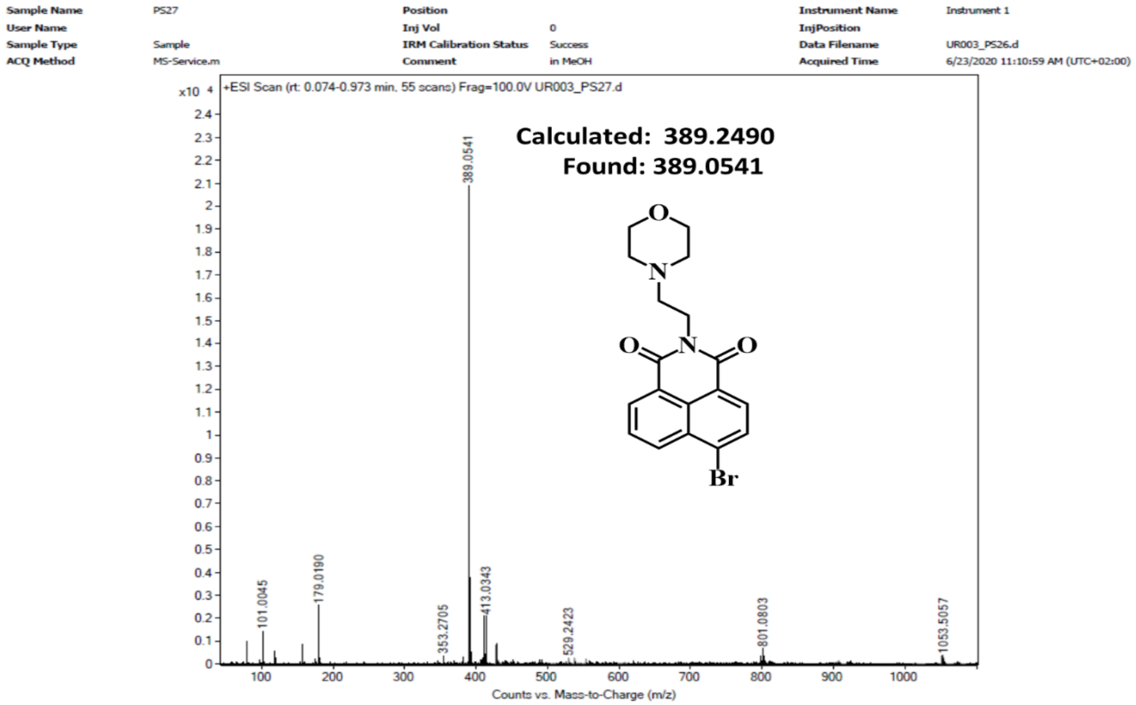

Figure S 3. ESI-MS spectra of compound **2** in methanol.

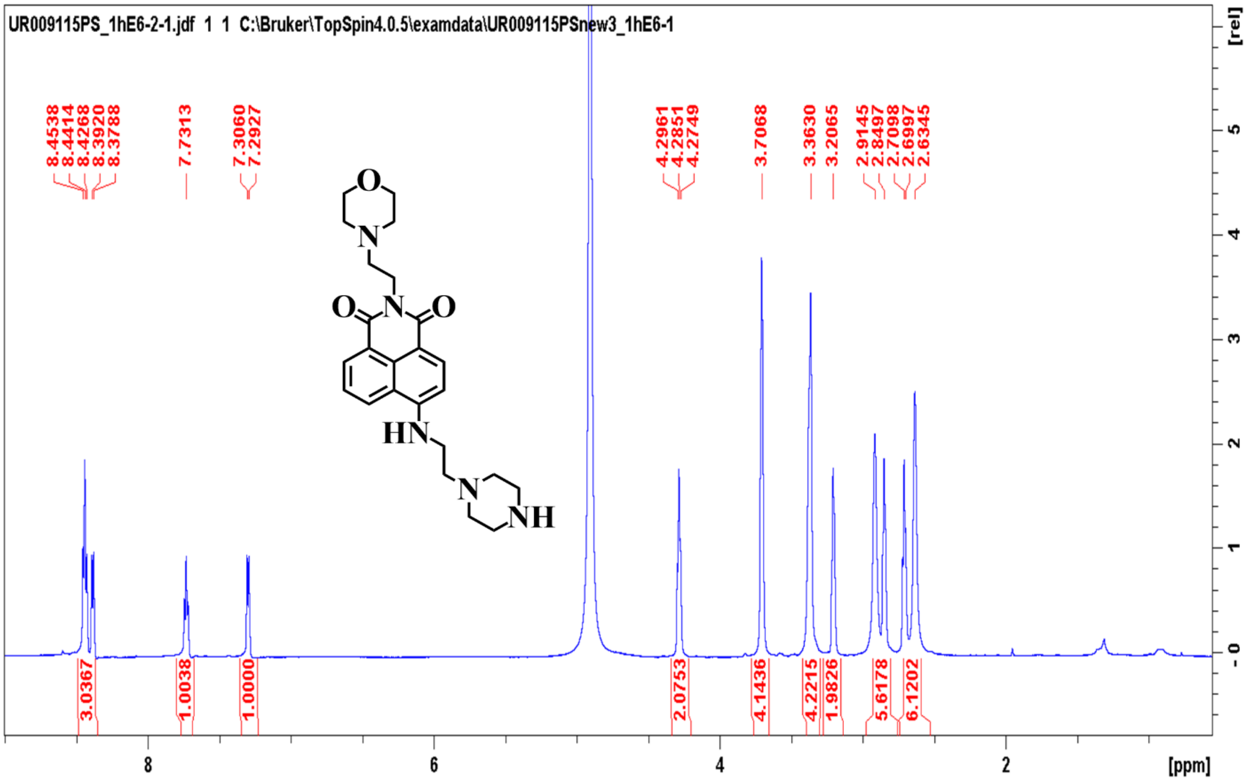

Figure S 4. ^{1}H NMR spectra of pH probe **3** in CD_3OD**.**

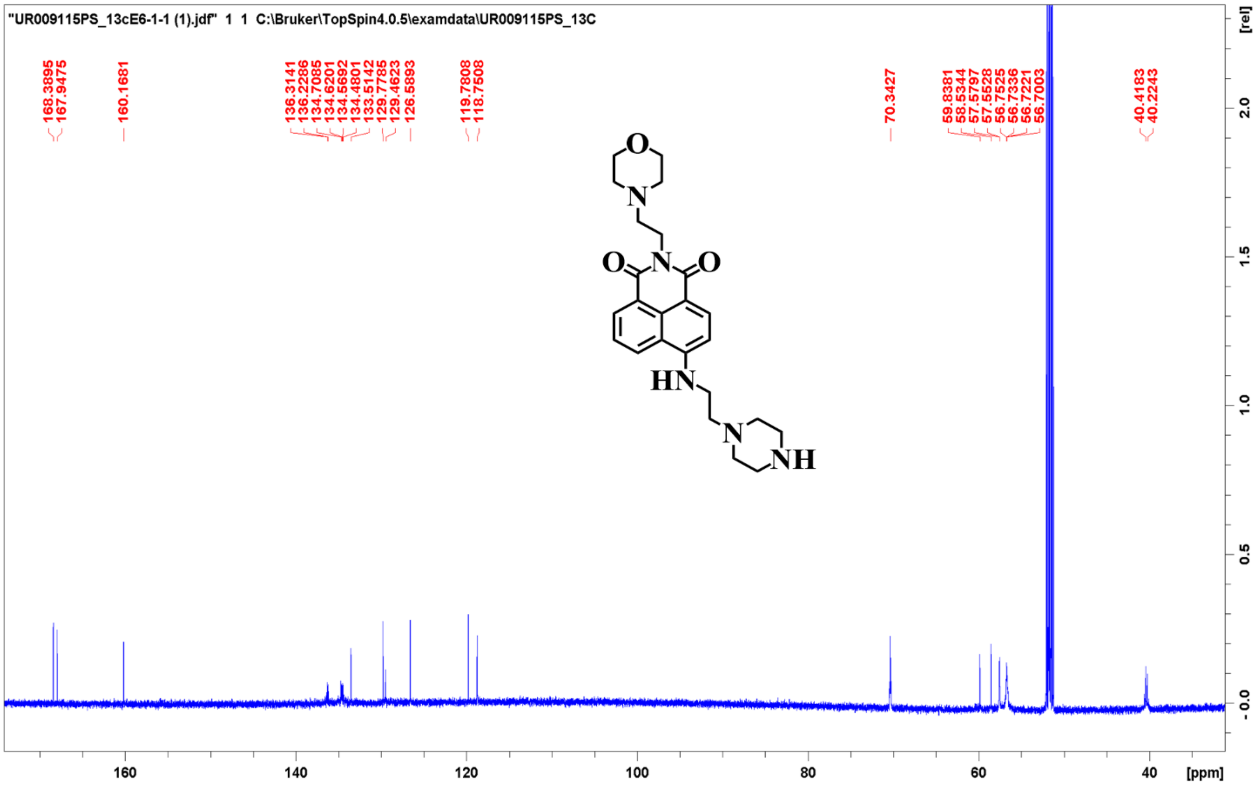

Figure S 5. ^{13}C NMR spectra of **3** in CD_3OD**.**

**Sample Name**	101PS	**Position**		**Instrument Name**	Instrument 1
**User Name**		**Inj Vol**	0	**InjPosition**	
**Sample Type**	Sample	**IRM Calibration Status**	Success	**Data Filename**	UR009_101PS.d
**ACQ Method**	MS-Service.m	**Comment**	in MeOH	**Acquired Time**	12/21/2021 8:24:10 AM (UTC+01:00)

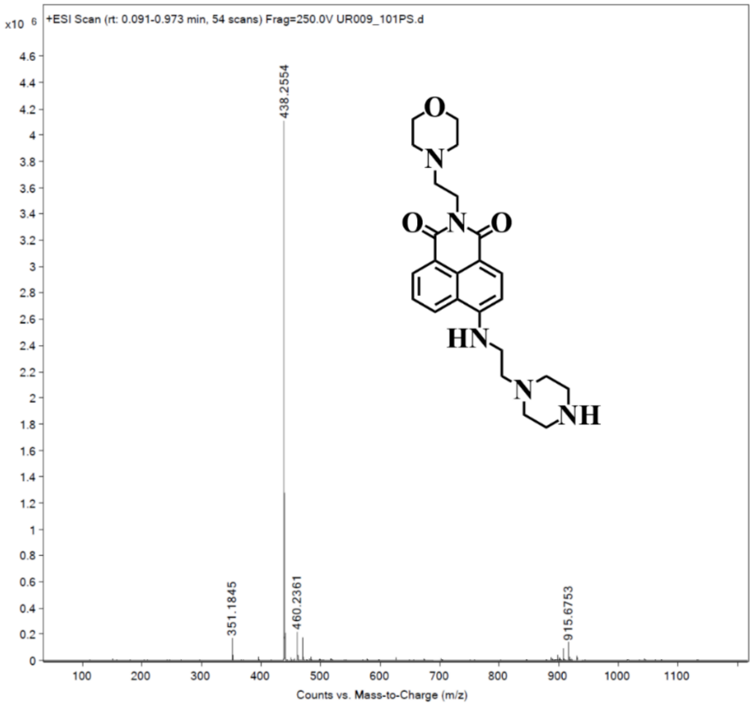

Figure S 6. ESI-MS spectra of **3** in methanol.

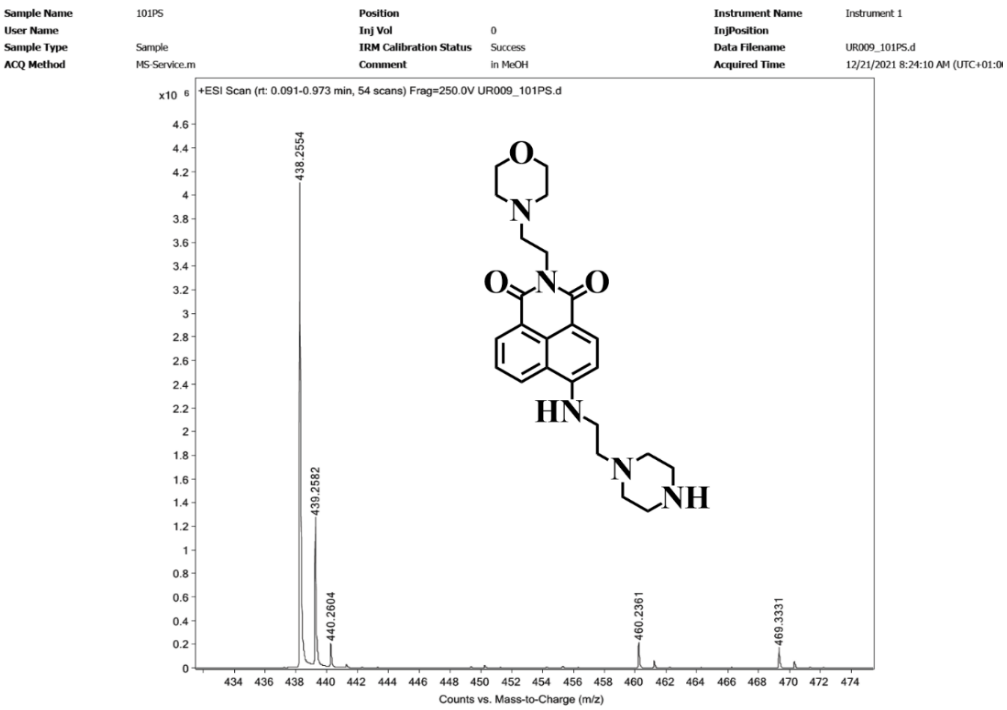

Figure S 7. Magnified ESI-MS spectra of **3** in methanol.

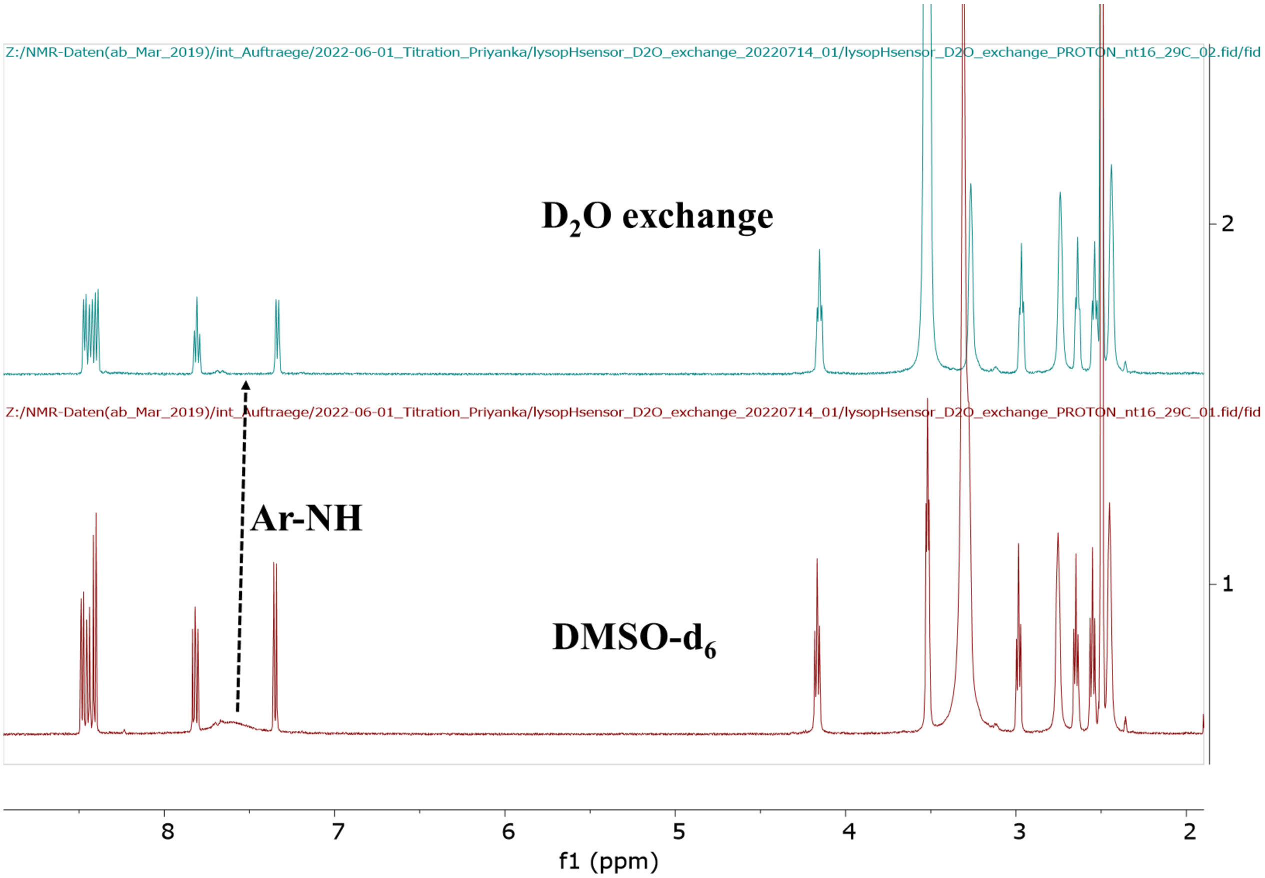

Figure S 8. ^{1}H NMR spectra of **3** in DMSO-d_6 and followed by D_2O exchange.

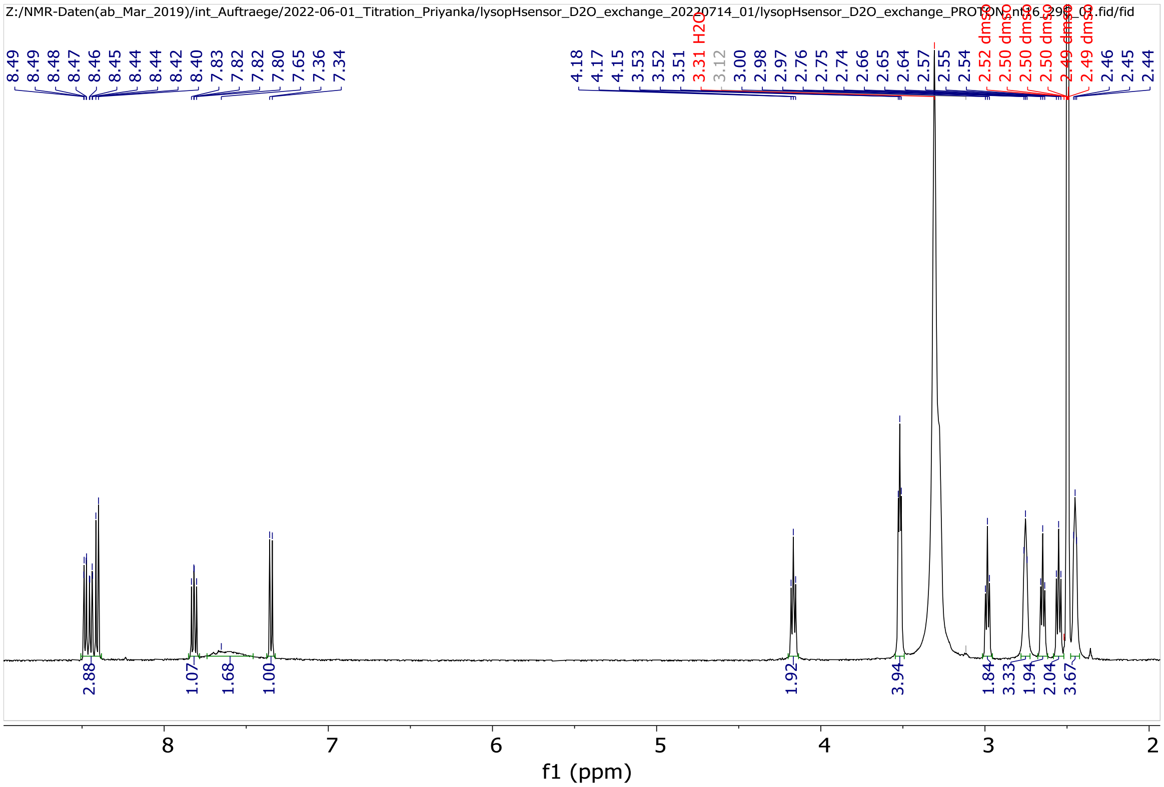

Figure S 9. ^{1}H NMR spectra of **3** in DMSO-d_6 (Figure S8 lower spectra).

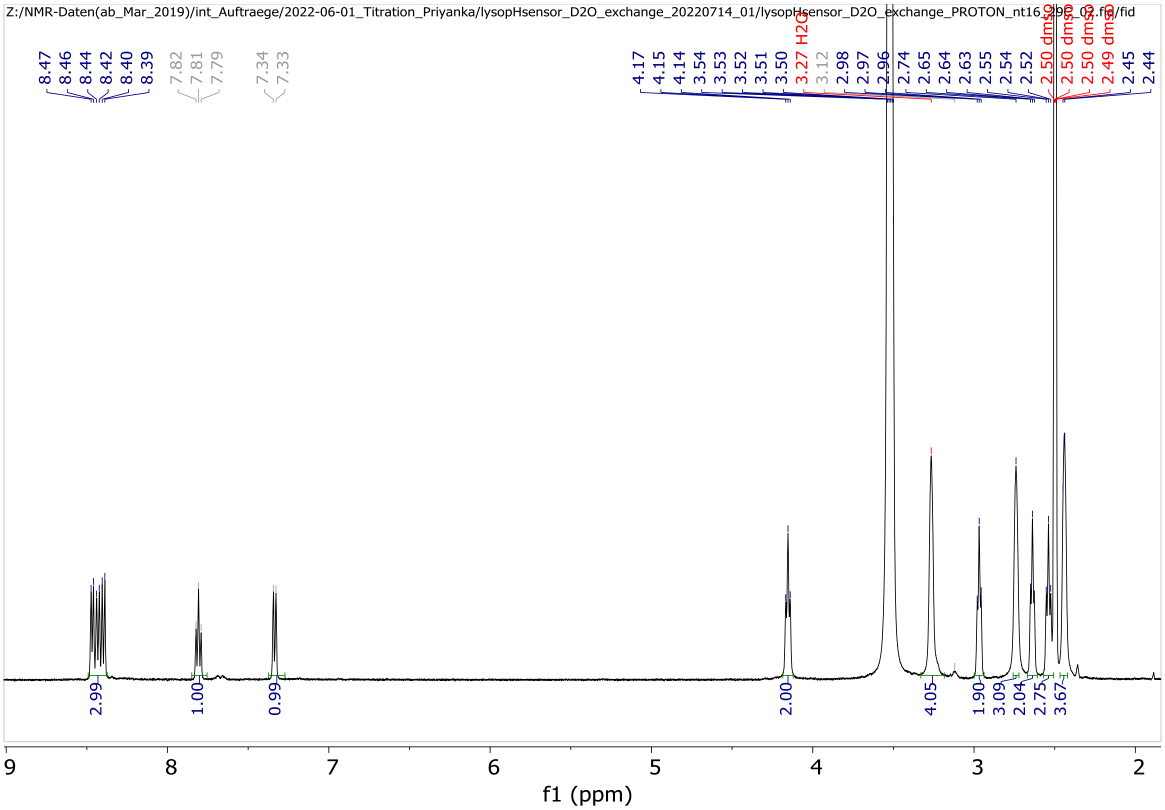

Figure S 10.^{1}H NMR spectra of **3** in DMSO-d_6 and followed by D_2O exchange (Figure S8 upper spectra).

To study the pH signaling mechanism of **3** in more detail, pH titration by NMR measurements in D_2O was performed. The pH probe was dissolved in D_2O and measured by addition of varying amounts of HCl, to change the pH from 8.5 to 2.5 (Figure S 11).

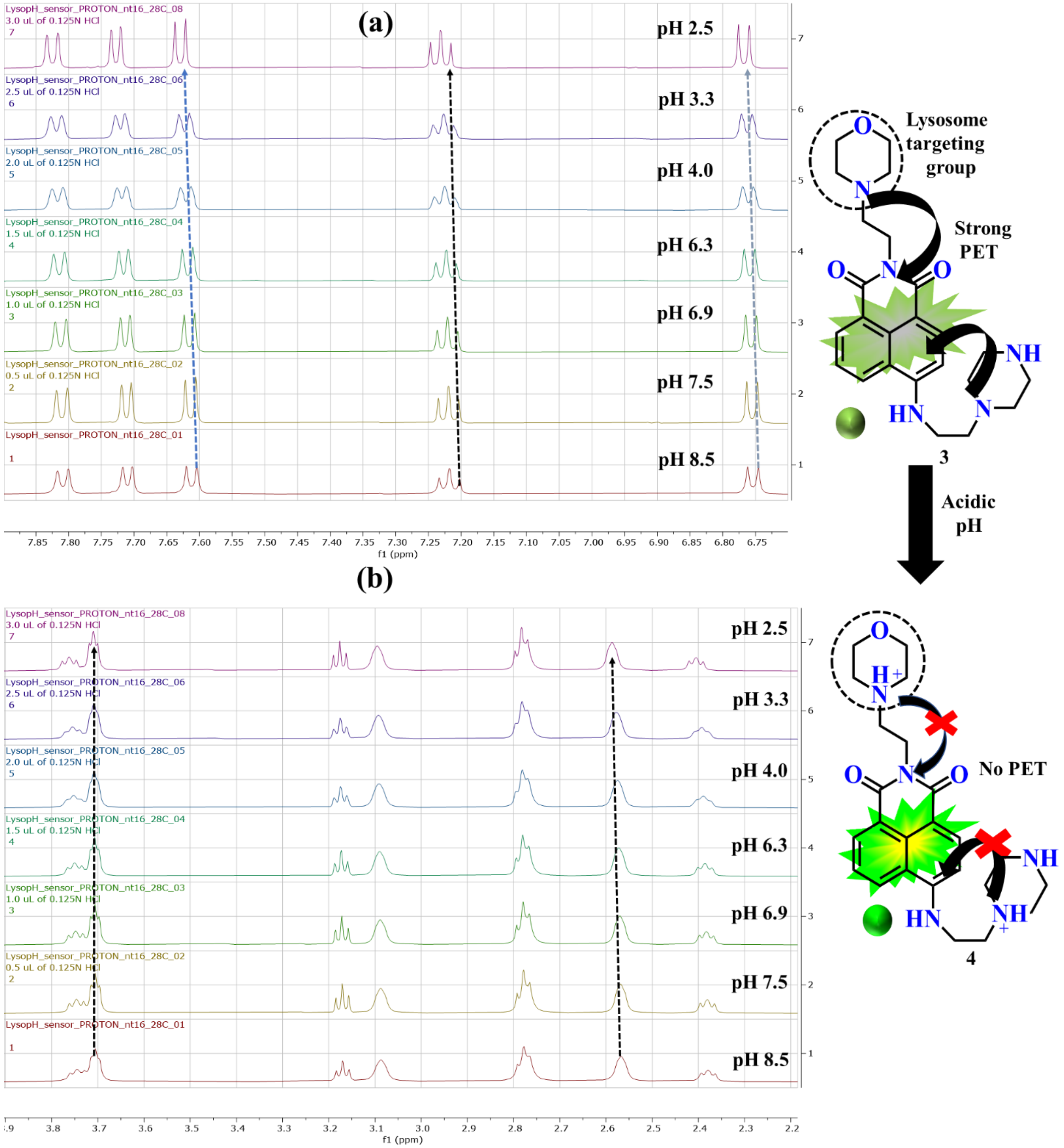

Figure S 11. NMR titration spectra of pH probe **3** performed in D_2O solvent and over a pH range of pH 8.5 to pH 2.5; (a) aromatic region (b) aliphatic region.

2. Optical spectroscopy of pH probe 3

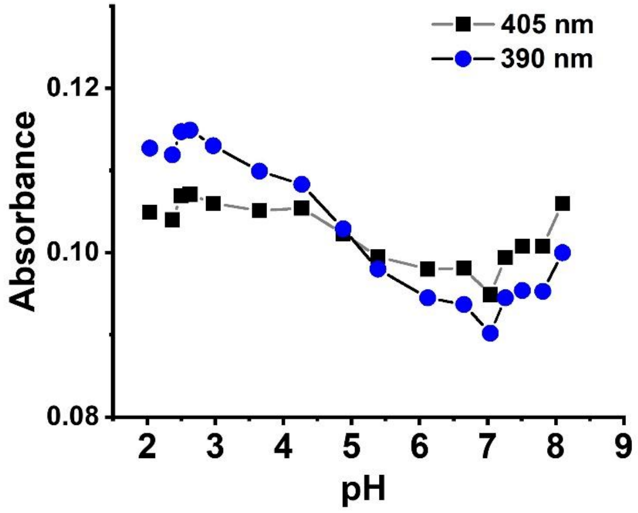

Figure S 12. Variation of absorbance (λ_{max} 405 nm and 392 nm) with different pH values of **3** in the pH range of 3.0 to 8.5 in water (Britton-Robinson buffer (B-R buffer)).

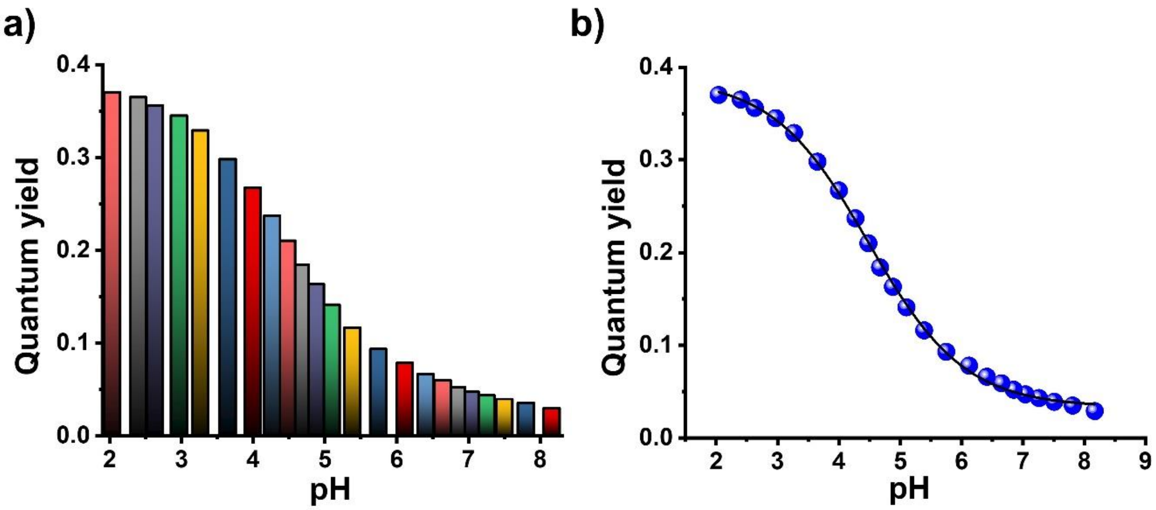

Figure S 13. Quantum yield of pH probe **3**; a) Bar diagram and b) plot of quantum yield at respective pH in the pH region 8.1 to 2.0 in an aqueous buffer (B-R buffer 25 mM).

Table S1. Quantum yield of **3** at different pH values; absorption maxima (λ_{max}, Abs), emission maxima (λmax, Em), and fluorescence quantum yields (Φ).

Sample	Solvent	Buffer	$\lambda_{max/\ Abs}$ [nm]	$\lambda_{max/\ Em}$ [nm]	pH	Φ	pH	Φ
**3**	MilliQ-water	B-R buffer	405	530	8.1	0.029	4.8	0.163
					7.8	0.035	4.6	0.184
					7.5	0.039	4.4	0.210
					7.2	0.043	4.2	0.237
					7.0	0.047	4.0	0.267
					6.8	0.052	3.6	0.298
					6.6	0.059	3.2	0.329
					6.4	0.066	2.9	0.345
					6.1	0.078	2.6	0.356
					5.7	0.093	2.4	0.365
					5.3	0.116	2.0	0.370
					5.1	0.141		

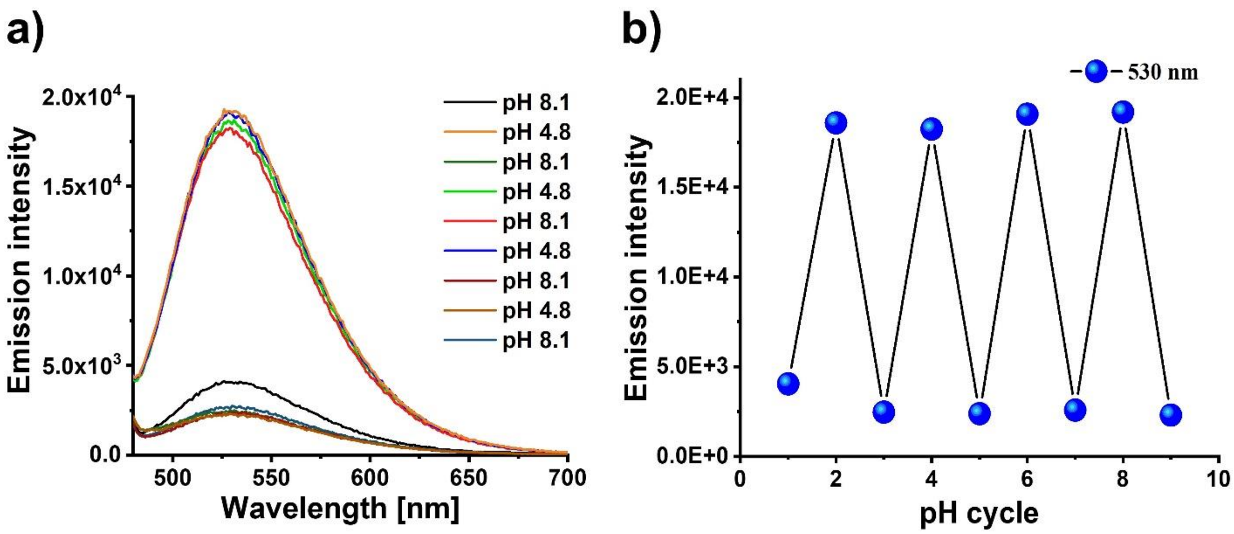

Figure S 14. Reversibility experiment of **3**; a) emission spectra and b) plot of emission intensity at 530 nm with varying pH upto three cycles from pH 8.1 to pH 4.8 in an aqueous buffer (B-R buffer 25 mM).

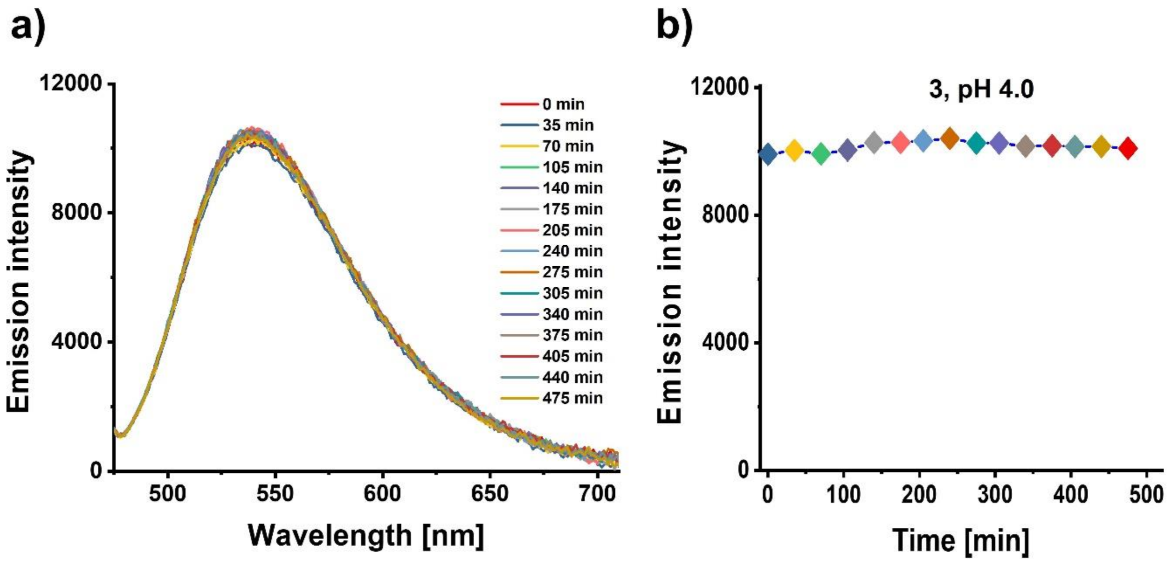

Figure S 15. Photostability experiment of **3** at pH 4.0; a) emission spectra and b) plot of emission intensity at 530 nm with increasing time upto 475 min (measured after every 35 min) in an aqueous buffer (B-R buffer 25 mM).

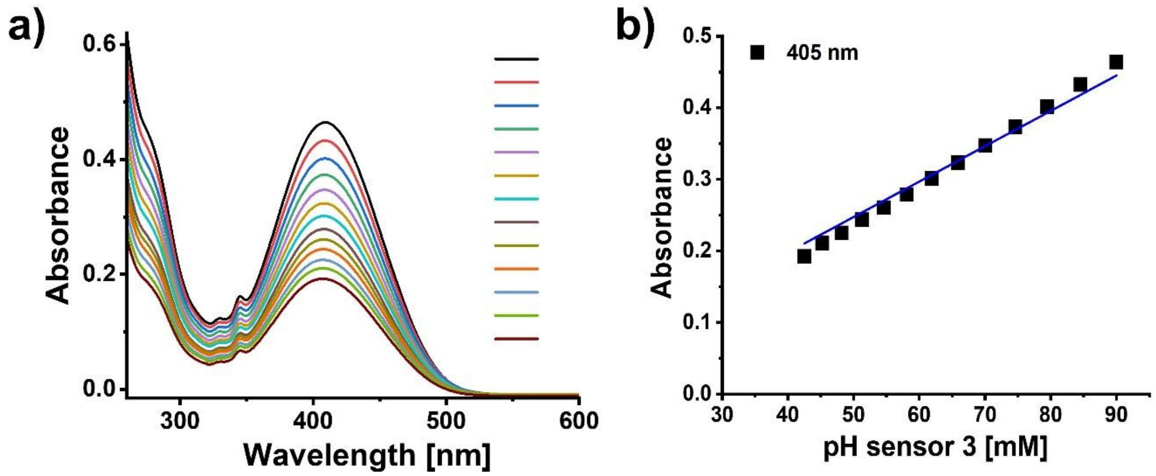

Figure S 16. a) Absorption spectra and b) calibration curve of **3** in water at different concentrations.

3. Characterization of PSNPs and SiO_2-NPs

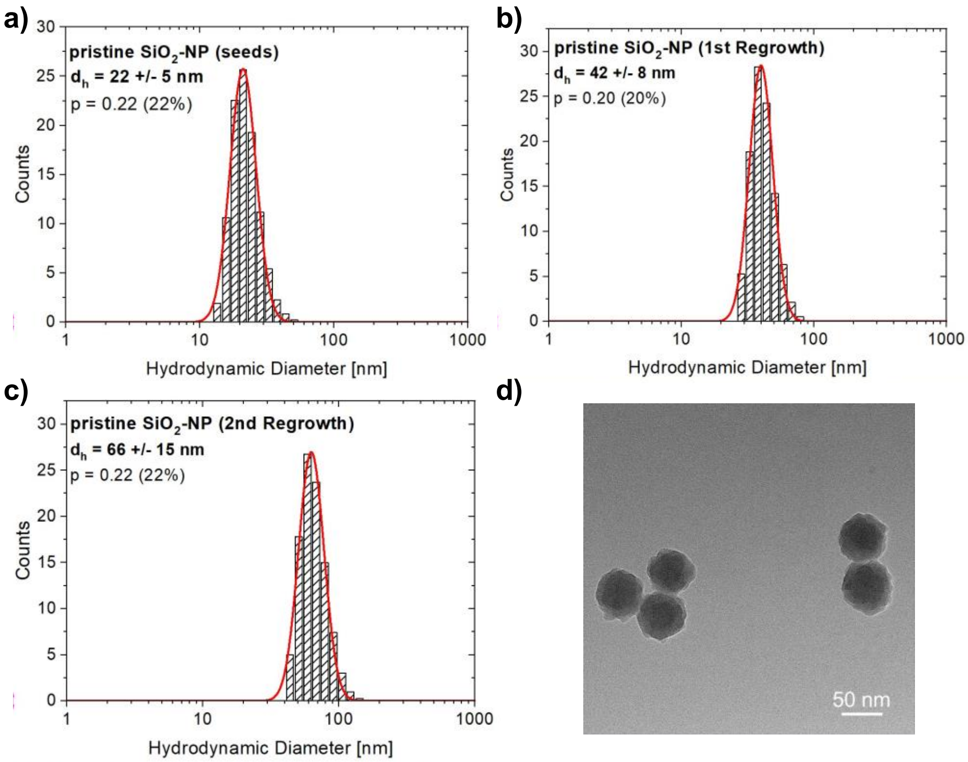

Figure S 17. Results of DLS measurements by number distribution of pristine SiO_2-NPs, after the formation of the seeds (a), after the first regrowth step (b), and after the second regrowth step (c), indicating a particle growth of around 20 nm in each step. TEM micrograph of pristine SiO_2-NPs after the second regrowth step showing spherical particles with a particle diameter of d_{TEM} = 60.5 ± 1.6 nm (d).

3.1 Synthesis of reference dyes: Silane derivatives of Nile Red (NR-Silane) and rhodamine B (RhB-APTES) and their embedding into the SiO_2-NPs

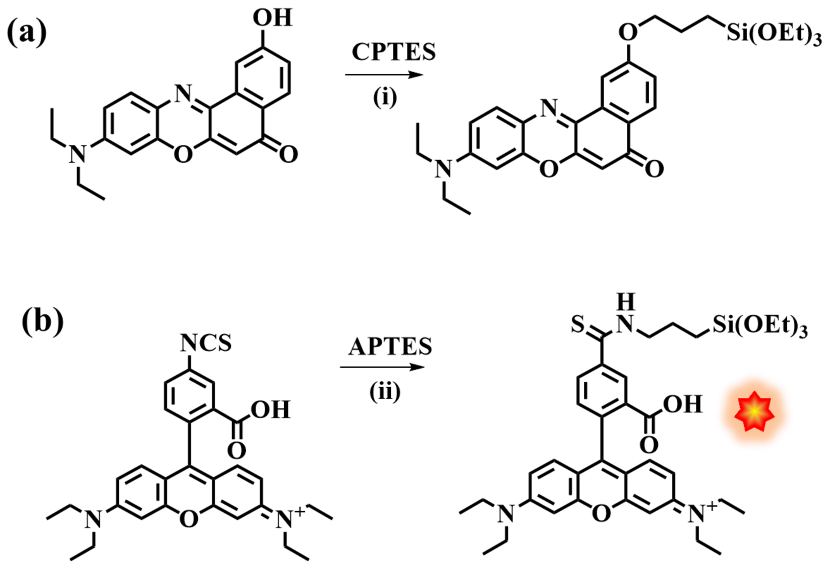

Figure S 18. Synthesis of NR-Silane derivative a) and RhB-APTES b); (i) K_2CO_3, DMF, 80 °C; (ii) ethanol, Ar, 2 d, room temperature (r.t.).

Synthesis of 9-Diethylamino-2-(triethoxysilyl-3-propyloxy)-5H-benzo[α]phenoxazin-5-one NR-Silane derivative (NR-Silane): 9-Diethylamino-2-hydroxy-5H-benzo[α]phenoxazin-5-one (Hydroxy nile red) was synthesized as reported in the literature (**Fehler! Verweisquelle konnte nicht gefunden werden.** a)).[1] Hydroxy Nile Red (20 mg, 0.05 mmol), K_2CO_3 (8.2 mg, 0.05 mmol) and 3-Chloropropylmethoxysilane (CPTES, 17.2 µL, 0.07 mmol) were taken in DMF. The reaction mixture was heated at 80 °C overnight. After complete reaction the solvent was removed and precipitate obtained was washed with hexane, dried and used without further purification.

Synthesis of reference dye RhB-APTES. The synthesis of RhB-APTES was adopted from the literature (**Fehler! Verweisquelle konnte nicht gefunden werden.** b)).[2] 9.97 mg (0.019 mmol) of RITC was dissolved in 1 mL of ethanol (absolute), followed by the addition of 13.2 µL (0.056 mmol, 2.9 equiv.) of 3-Aminopropyltriethoxysilane (APTES) under a strict argon atmosphere; the resulting mixture was then allowed to stir for 2 d at room temperature (r.t.). Finally, the prepared RhB-APTES was stored under light exclusion at 4 °C, to be utilized later in dye embedding experiments.

Loading of SiO_2-NP with NR and NR-Silane. To obtain 80 nm large SiO_2-NPs loaded with NR or NR-Silane, the synthesis of the particles was performed as described for RhB-APTES loaded NPs, only in the last regrowth step 0.15 mL ($6.4 \cdot 10^{-7}$ mol) of NR or 0.15 mL ($6.4 \cdot 10^{-7}$ mol) of NR-APTES in DMSO was injected into the aqueous phase 20 min after the addition of TEOS.

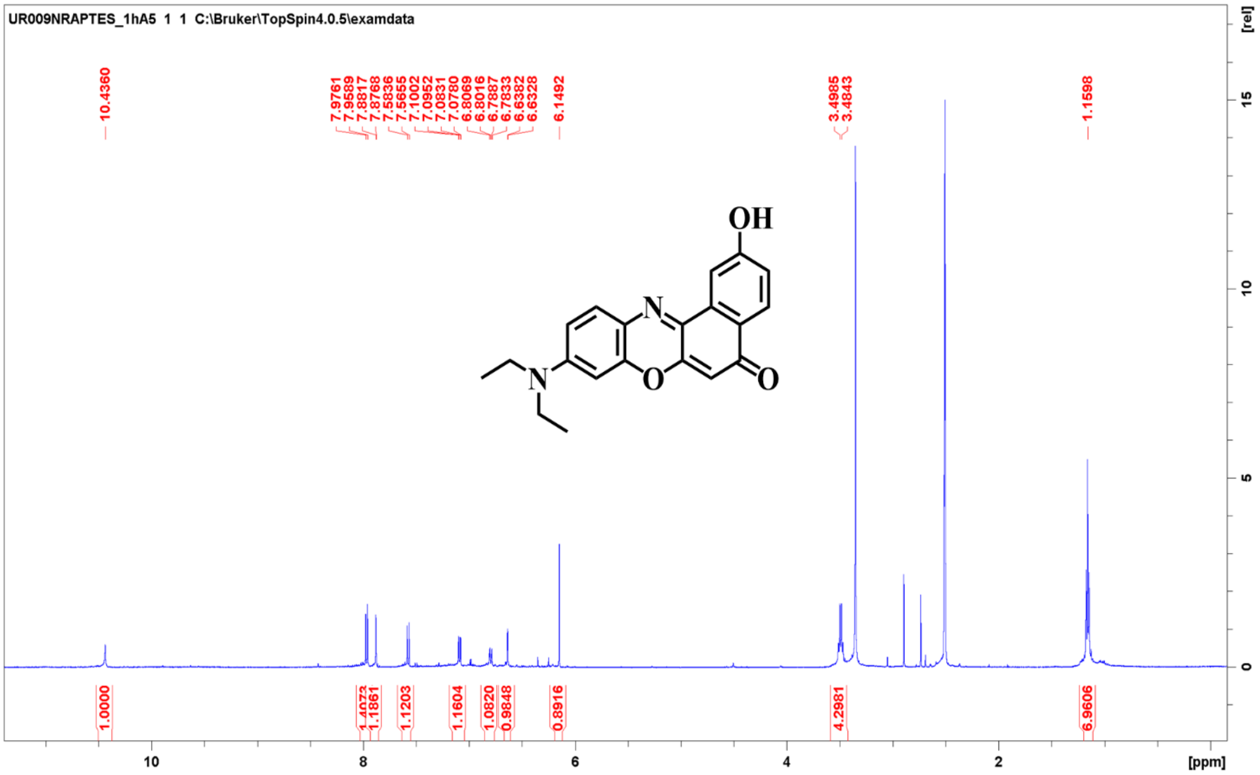

Figure S 19. ^{1}H NMR of Hydroxy derivative of nile red in DMSO-d_6.

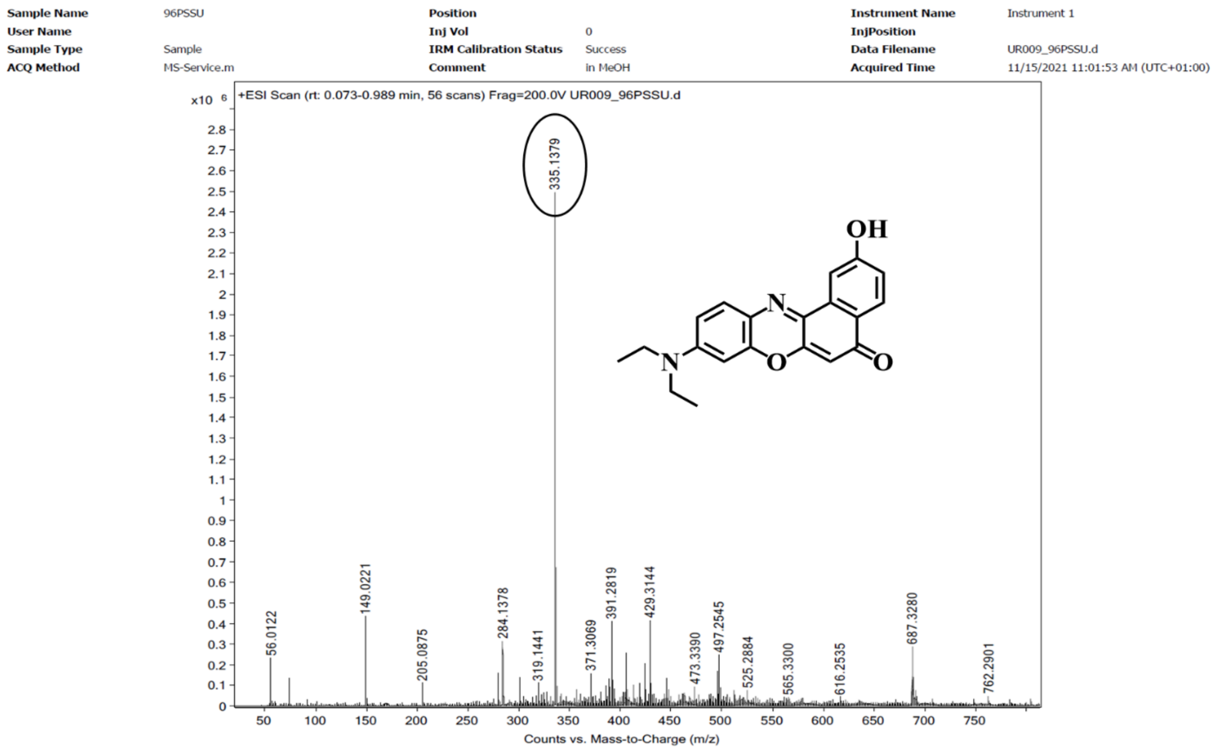

Figure S 20. ESI-MS spectra of Hydroxy derivative of Nile Red in MeOH.

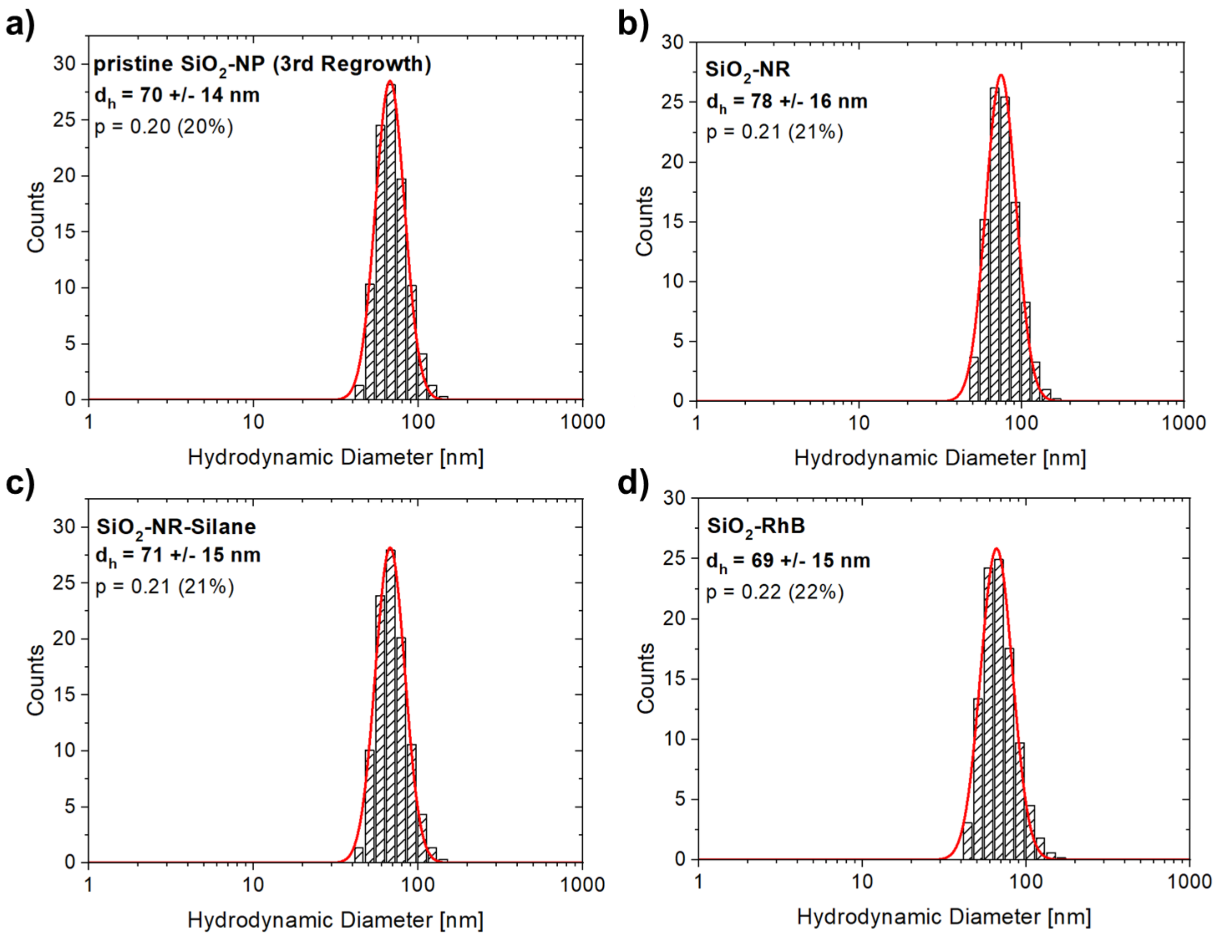

Figure S 21. Results of DLS measurements by number distribution of dye stained SiO_2-NPs, using Nile Red (NR) b), NR-Silane c), and RhB-APTES d).

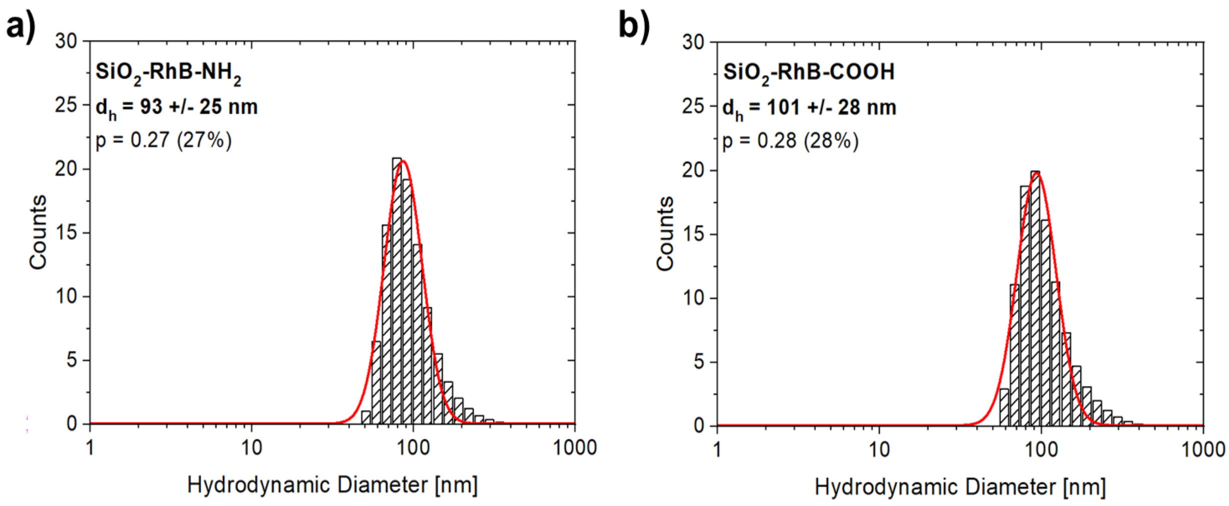

Figure S 22. Results of DLS measurements by number distribution of RhB-APTES stained SiO_2-NPs after surface modification with APTES a) and succinic anhydride b). The measured z-average are 164 ± 3 nm (SiO_2-RhB-NH_2) and 176 ± 1 nm (SiO_2-RhB-COOH), respectively.

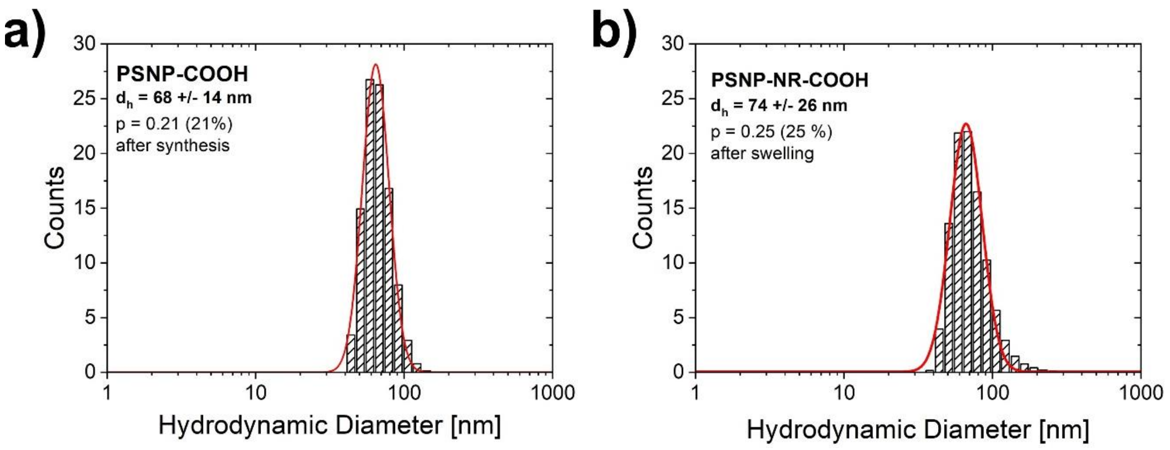

Figure S 23. Results of DLS measurements by number distribution of the pristine PSNPs after their synthesis a) and after the embedding of the NR dye by a swelling step (PSNP-NR-COOH, b)). The measured z-average are 96 ± 0.2 nm (PS-COOH) and 177 ± 7 nm (PSNP-NR-COOH), respectively.

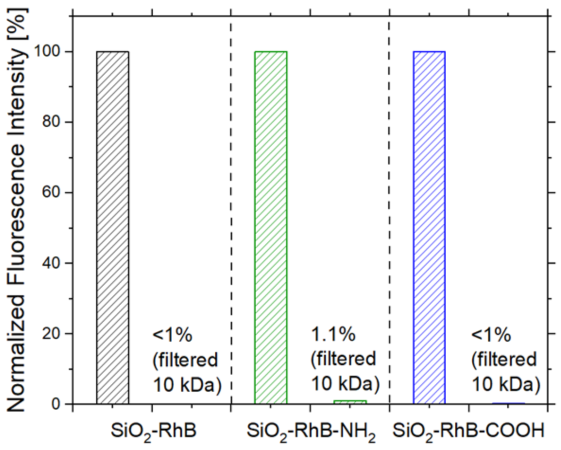

Figure S 24. Dye leaking experiments after encapsulation of RhB into the SiO_2 matrix and the influence of surface modification (λex = 520 nm).

3.2 Determination of the amount of reference dyes in the particle cores

To determine the amount of loaded reference dye molecules per particle a dissolution method was used. Different concentrations of NR in THF and RhB-APTES in aqueous B-R buffer were dissolved and the emission spectra (Figure S 25 and Figure S.27) and calibration curves were recorded. In addition, a previously dried amount of PSNP-NR-COOH of known mass was dissolved in 2.5 mL of THF and the emission spectrum was measured (Figure S 26), while SiO_2-RhB-COOH were dissolved in aqueous B-R buffer at pH 12. The amount of reference dye molecules per particle was then calculated from the experimentally determined (average) amount of incorporated dye and the number of particles in the dispersion, using the number-based hydrodynamic diameters and a density of $\rho(PS) = 1.06\ g/cm^3$ and $\rho(SiO_2) = 2.09\ g/cm^3$.

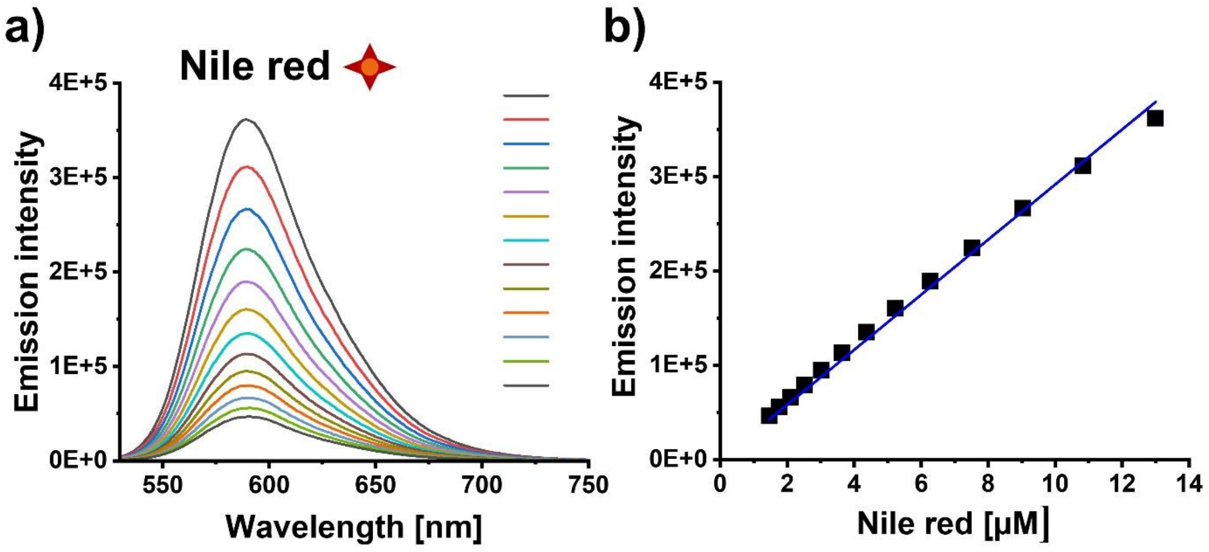

Figure S 25. a) Emission spectra and b) calibration curve of NR in THF at different concentrations.

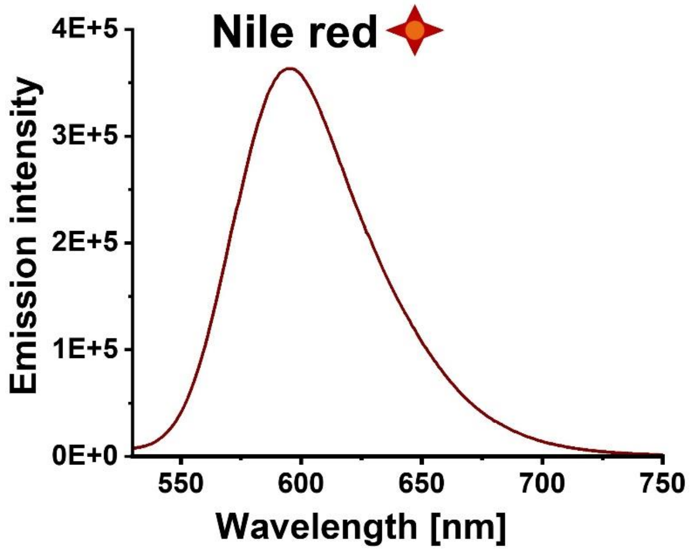

Figure S 26. Emission spectra of PSNP-NR-COOH after dissolving them in THF.

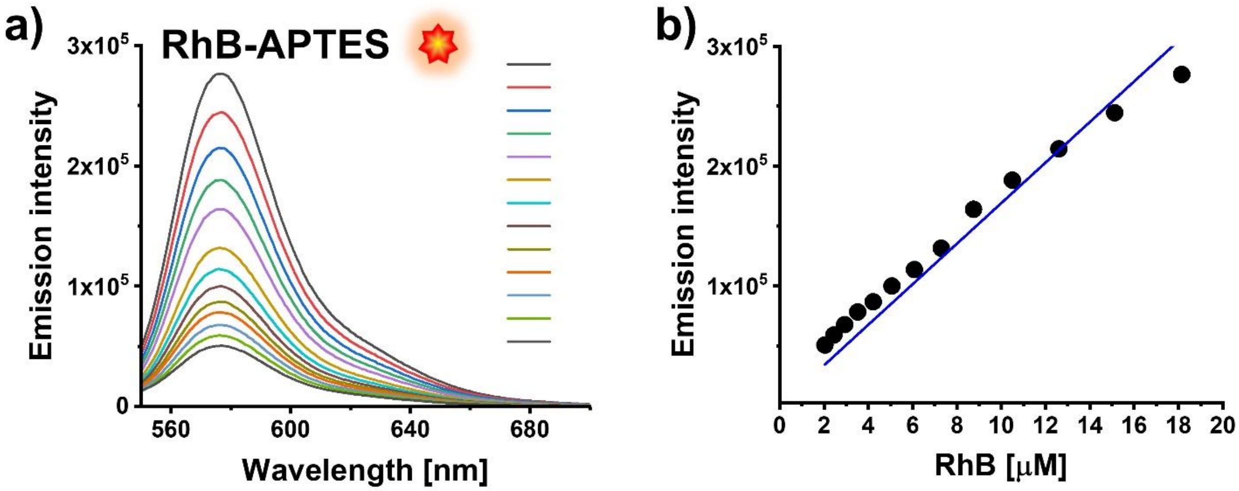

Figure S 27. a) Emission spectra and b) calibration curve of RhB-APTES at pH 12 in aqueous B-R buffer.

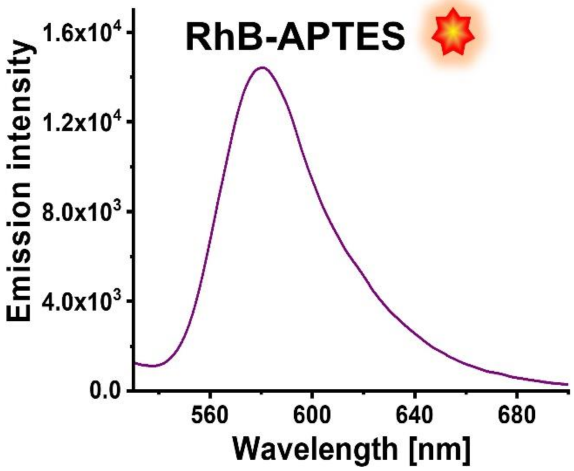

Figure S 28. Emission spectra of SiO_2-RhB-COOH at pH 12 in aqueous B-R buffer.

3.3 Determination of the number of total and accessible COOH groups on the particle surface of PSNP-NR-COOH and SiO_2-RhB-COOH

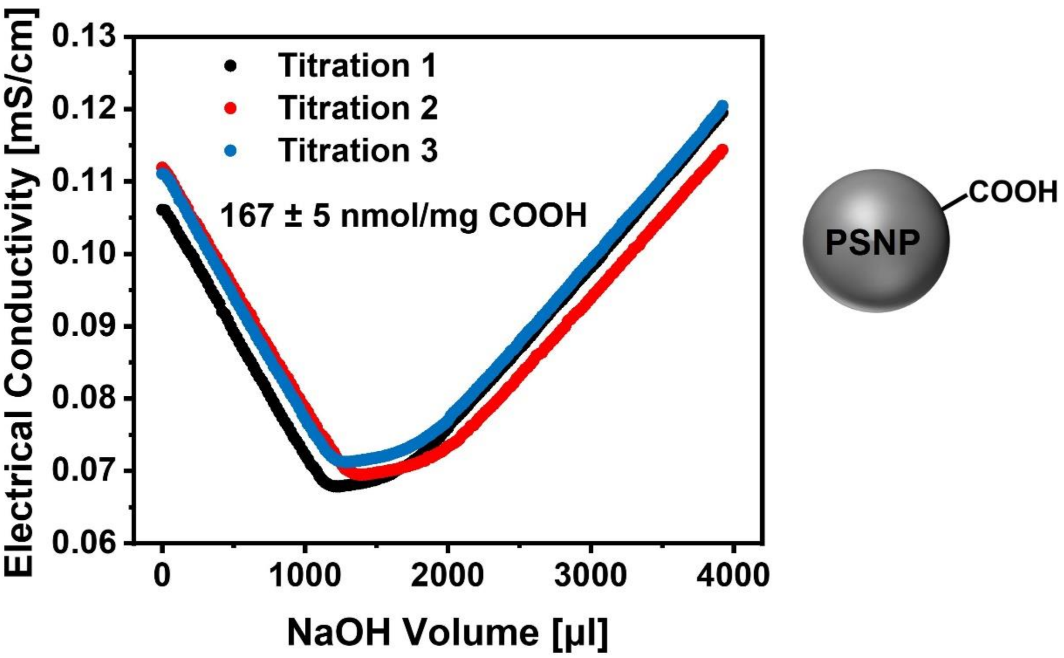

Figure S 29. Results of conductivity measurements of PSNPs.

To determine the accessible number of COOH groups on the particle surface a toluidine blue assay was performed.[3] Therefore, 0.2 mL of toluidine blue O in MilliQ water, containing 3.2 µmol of the dye, were added to 0.8 mL of the PSNP-NR-COOH in MilliQ water. After 15 min of gentle shaking, the particles were centrifuged (16,000 rcf/15 min) and the supernatant was collected. The absorbance spectra of the supernatant (175 µL & 1.325 µL of MilliQ water) and of a toluidine blue O solution with 3.2 µmol/1 mL (10 µL & 1.49 µL of MilliQ water) were recorded, and the absorbance values at the maximum (633 nm) were used in equation (1). The particle mass was determined to be 2.49 mg after the assay.

$$\boldsymbol{accessible\ COOH\ groups\ (PSNP - COOH) = \frac{abs(supernatant) \cdot n(TB\ solution)}{abs(TB\ solution) \cdot m(particles)}}$$
$$\boldsymbol{= \frac{0.3584 \cdot 3.2\ \mu mol}{17.5 \cdot 0.7493 \cdot 2.49\ mg} = 35\ \frac{nmol}{mg} \quad (1)}$$

Equation S1. Calculation of the number of accessible COOH groups on the surface of PSNP-NR-COOH, obtained by an optical toluidine blue O assay, leading to a functional group density of 35 nmol/mg.

In comparison, 0.2 µL (3.2 µmol) of toluidine blue O in MilliQ water were added to 0.4 mg of SiO_2-RhB-COOH in MilliQ water. After 15 min of incubation, the particles were washed by several cycles of centrifugation (15,000 rcf/20 min), removal of supernatant and addition of MilliQ water. The supernatants of all washing steps were collected. When the supernatant was clear, 1 mL of 1% SDS was added and the particles were incubated for another 30 min with gentle shaking. After centrifugation, an absorption spectrum (633 nm) of the supernatant was measured to determine the amount of desorbed toluidine blue O. Finally, the particles were extensively washed to remove SDS, dried *in vacuo* and the weight of the remaining particles was determined.

3.4 Surface modification of carboxylated PSNPs and SiO_2-NPs with 3

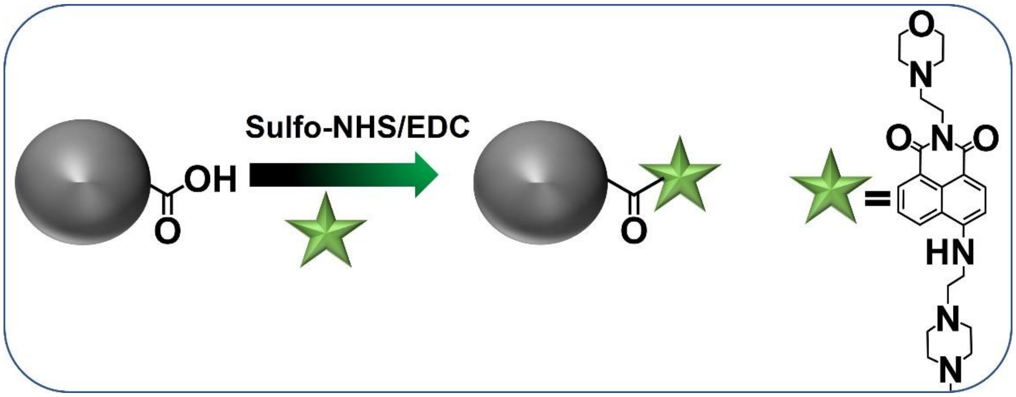

Figure S 30. Reaction scheme of the surface modification of plain, carboxylated PSNPs (no red reference dye in the particle core) with **3**.

IR measurements

FT-IR measurements were measured on Vertex 70 instrument from Bruker with MCT (N_2(l) cooled) detector. To confirm the binding of the synthesized optical probe **3** on the surface of the particles, PSNP-COOH with only 3 on the particle surface is synthesized as shown in Figure S 30and compared by measuring the FT-IR spectra of PSNP-COOH, free **3** and PSNP-**3** in KBr pellets. pH sensor **3** showed bands at 2923 cm^{-1}, 1692 cm^{-1}, 1614 cm^{-1}, 1513 cm^{-1}, 1383 cm^{-1}, 1234 cm^{-1} and 1040 cm^{-1} corresponding to the stretching vibrations of CH, CO, aromatic -C=C, C-C-O, -C-N in the IR spectra. The spectra of carboxylated PSNPs showed bands at 3024 cm^{-1}, 2922 cm^{-1} and 2847 cm^{-1}, corresponding to the stretching vibrations of aromatic CH, and CH/CH_2, respectively. The bands at 1599 cm^{-1}, 1492 cm^{-1}, 1068 cm^{-1} and 1027 cm^{-1} are due to the stretching vibration of aromatic C=C, CH_2, C-C respectively. The PSNP-**3** showed 1027 cm^{-1}, 1218 cm^{-1}, 1741 cm^{-1}, 1661 cm^{-1}, 1371 cm^{-1} bands corresponding to the C-O and C-N stretching similar to **3** besides the stretching observed in the PSNPs indicating the binding of the sensor on the surface of the particles.

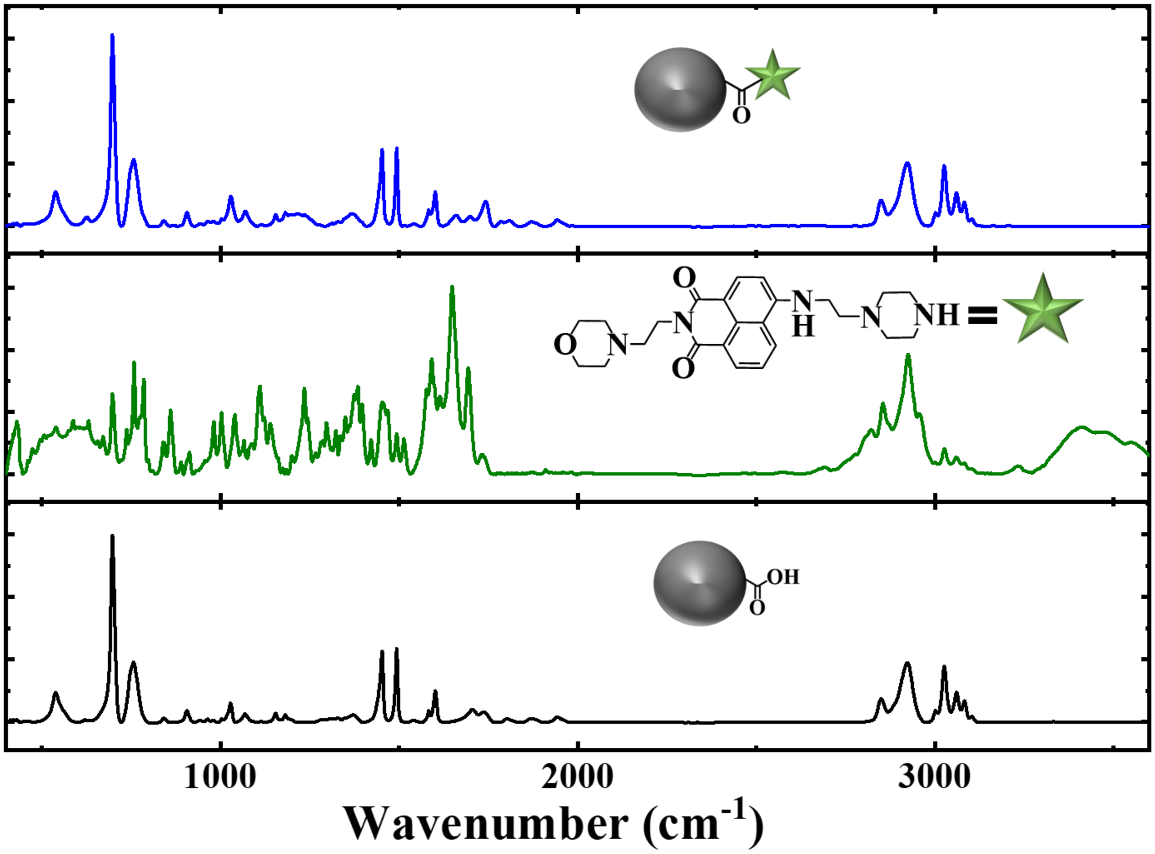

Figure S 31. FT-IR spectra of PSNP-COOH (black), free **3** (green) and PSNP-**3** (blue) measured on KBr pellets.

4. Optical spectroscopy of nanosensors

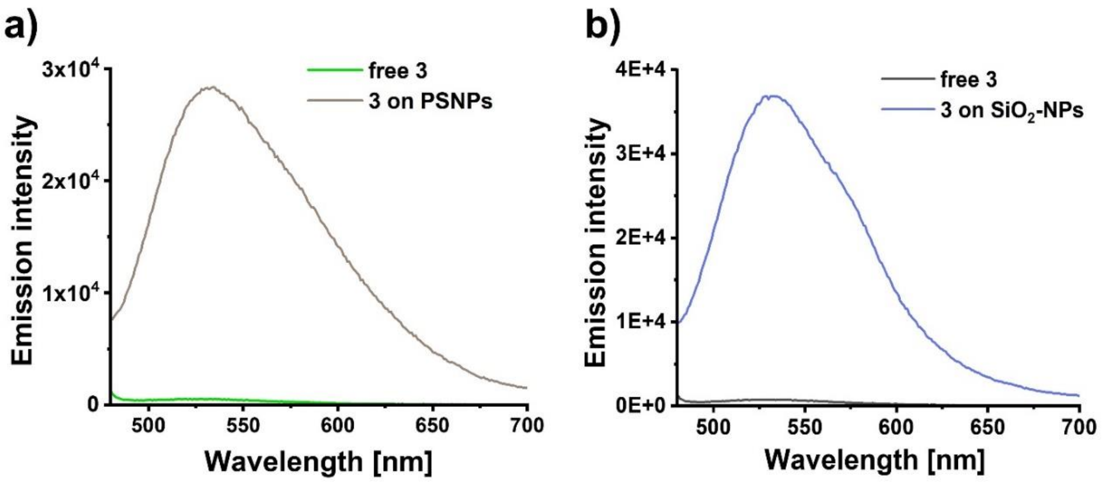

Figure S 32. Emission spectra of free **3** in the supernatant and bound **3** on the particles surface of PSNPs (a)) and SiO_2-NPs (b)) after dialysis.

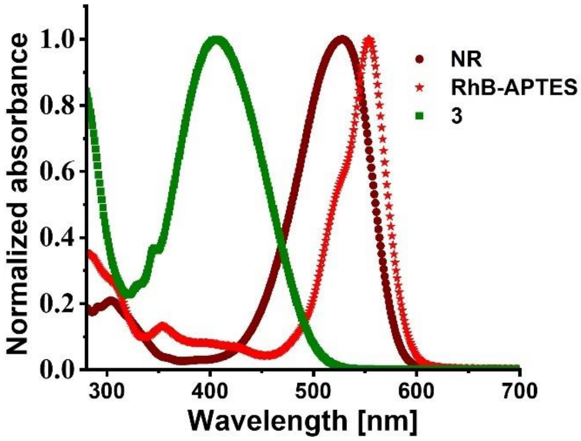

Figure S 33. Normalized absorbance spectra of the reference dyes NR and RhB-APTES as well as the free optical probe 3 in THF (NR) or an aqueous buffer (B-R buffer 25 mM, RhB-APTES, 3).

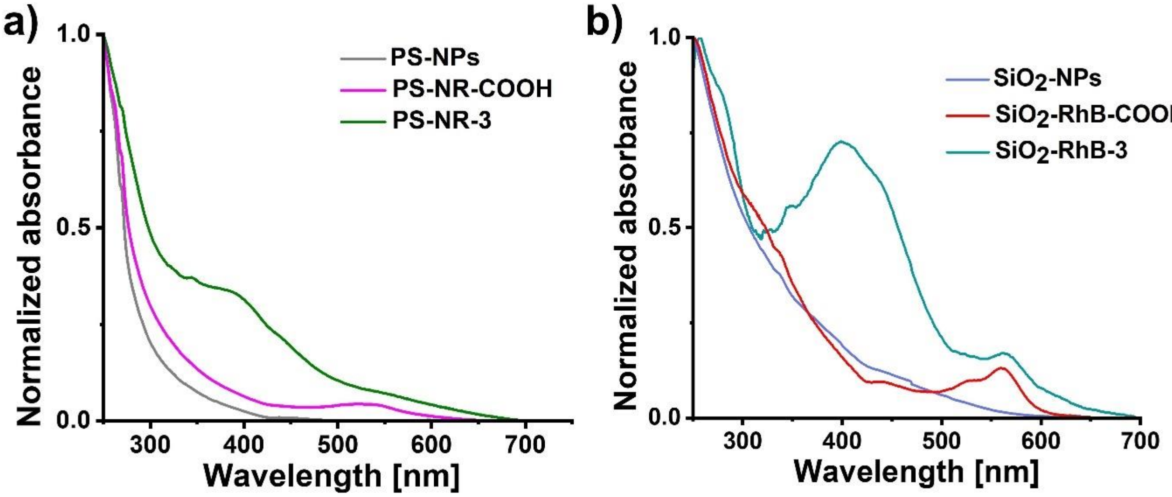

Figure S 34. Normalized absorption spectra of a) PSNPs (plain polystyrene particles with COOH groups), PSNP-NR-COOH (PSNPs with encapsulated NR) and PSNP-NR-**3;** (b) pristine SiO_2-NPs, SiO_2-RhB-COOH (silica particles with encapsulated RhB and COOH groups on the particle surface) and SiO_2-RhB-**3** in an aqueous buffer (B-R buffer 25 mM).

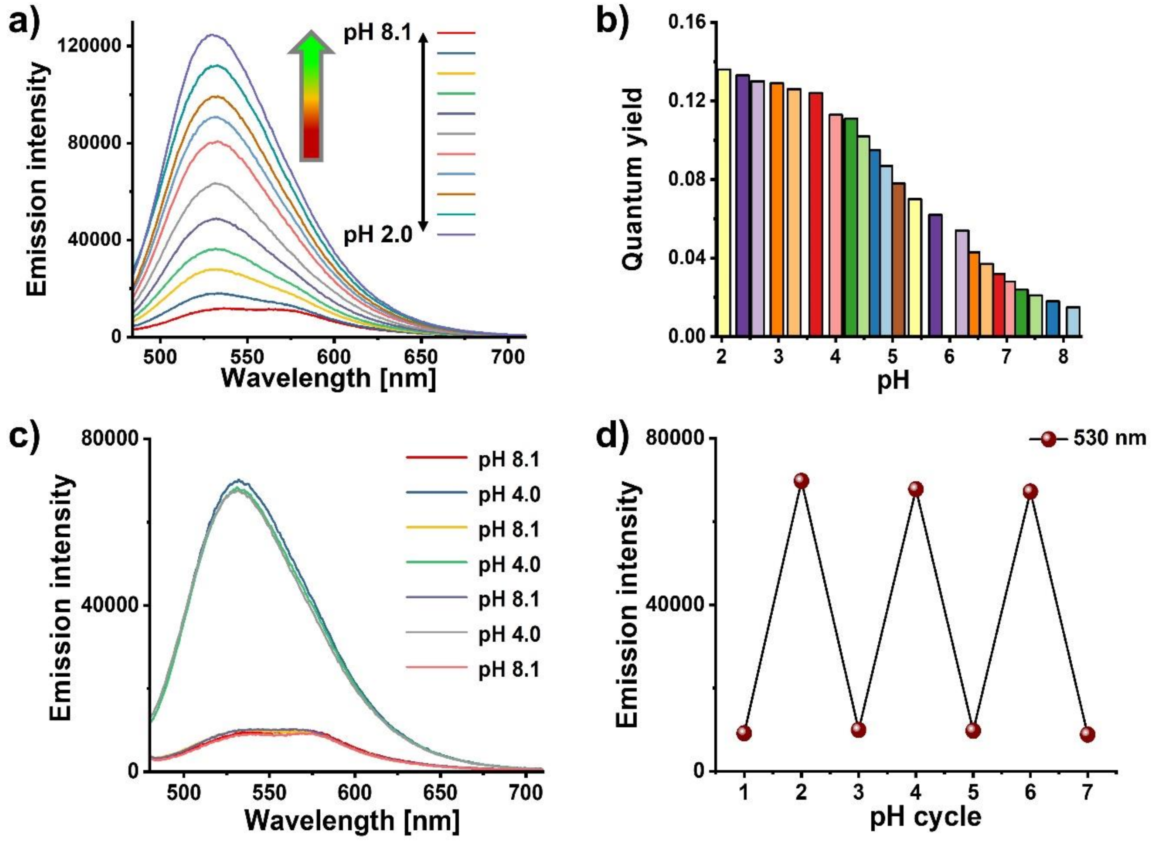

Figure S 35. pH-dependent fluorescence of SiO_2-RhB-**3**. a) Emission spectra at different pH values; b) bar diagram of the pH-dependence of quantum yields in the pH range of 8.1 to 2.0; c) and d) Reversibility study of SiO_2-RhB-**3** involving the measurement of the emission spectra and the fluorescence intensity by varying the pH from 8.1 to 4.0 in cycles in an aqueous B-R buffer (25 mM).

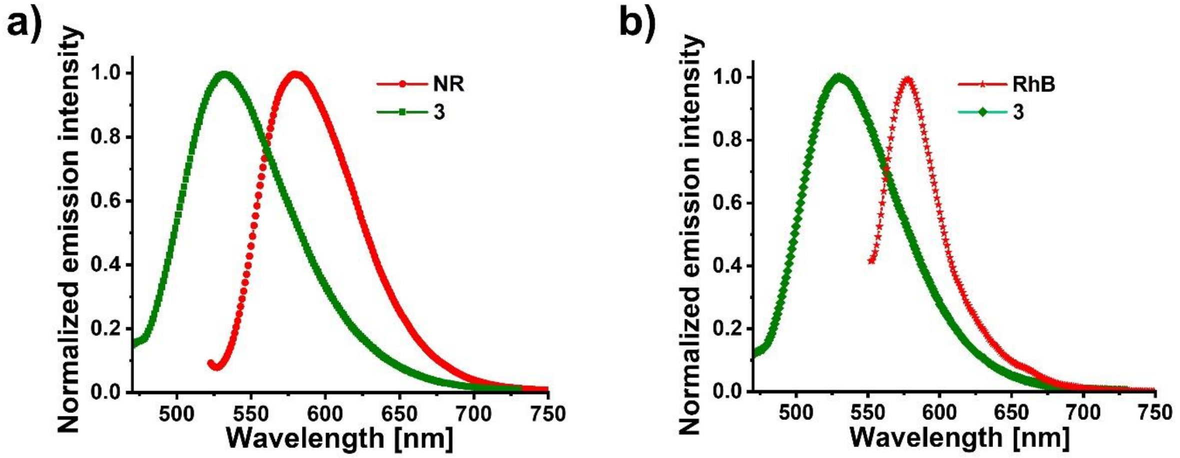

Figure S 36. Normalized emission spectra of particles with **3** and reference dyes NR and RhB-APTES at pH 4; a) NR (λ_{Ex} 510 nm) and **3** (λ_{Ex} 405 nm) in PS-NR-**3**; b) RhB-APTES (λ_{Ex} 540 nm) and **3** (λ_{Ex} 405 nm) in SiO_2-RhB-**3** in an aqueous B-R buffer (25 mM).

Table S2. Quantum yield of PSNP-NR-**3,** at different pH values, absorption maxima (λ_{max}, Abs), emission maxima (λ_{max}, Em), and fluorescence quantum yields Φ in an aqueous B-R buffer.

Sample	Solvent	Buffer	λ max/ Abs [nm]	λ max/ Em [nm]	pHs	Φ	pHs	Φ
PS-NR-**3**	MilliQ water	B-R buffer	400 and 538	533 and 578	8.1	0.032	4.8	0.081
					7.8	0.036	4.6	0.086
					7.5	0.038	4.4	0.088
					7.2	0.044	4.2	0.092
					7.0	0.049	4	0.096
					6.8	0.052	3.6	0.1
					6.6	0.055	3.2	0.107
					6.4	0.062	2.9	0.121
					6.1	0.064	2.6	0.125
					5.7	0.071	2.4	0.137
					5.3	0.072	2.0	0.134
					5.1	0.078		

Table S3. Quantum yield of SiO_2-RhB-**3,** at different pH values, absorption maxima (λ_{max}, Abs), emission maxima (λ_{max}, Em), and fluorescence quantum yields Φ in an aqueous B-R buffer.

Sample	Solvent	Buffer	λ max/ Abs [nm]	λ max/ Em [nm]	pHs	Φ	pHs	Φ
Si-RhB-**3**	MilliQ water	B-R buffer	400 and 560	530 and 577	8.1	0.015	4.8	0.087
					7.8	0.018	4.6	0.095
					7.5	0.021	4.4	0.102
					7.2	0.024	4.2	0.111
					7.0	0.028	4	0.113
					6.8	0.032	3.6	0.124
					6.6	0.037	3.2	0.126
					6.4	0.043	2.9	0.129
					6.1	0.054	2.6	0.13
					5.7	0.062	2.4	0.133
					5.3	0.07	2.0	0.136
					5.1	0.078		

5. Fluorescence cell microscopy

Cellular fluorescence microscopy

The human lung cancer cell line A549 were routinely propagated as described in the literature. [4] In short, the , A549 cells were cultivated in DMEM medium, to which 10% fetal calf serum (FCS), 2% glutamine, and penicillin / streptomycin (purchased from PAN Biotech) were added. Cells were disseminated into medium at a concentration of 1 x 10^5 cells/mL, cultivated at 37 °C with 5% CO_2, and split 1:5 twice per week. Using a 24-well culture plate prepared with glass cover slips (Sigma Aldrich), cells were disseminated at a concentration of 1 x 10^5 cells/mL in 1 mL of medium and cultured at 37 °C and 5% CO_2 for 48 h. Cells were rinsed with sterile PBS, after which cell pH was adjusted following the protocol by Lucien *et al.*[5], with pH values of 4.5, 5.5, and 7.5 being used over time periods of 30 min, 1 h, 3 h, or 24 h. Nanoparticle samples (100 µg/mL) and free pH probe **3** (35 µg/mL) in sterile PBS were added to the cells and incubated for 30 min at 37 °C and 5% CO_2. Cells were rinsed again using sterile PBS and fixed with cold PFA solution. 4,6-diamidino-2-phenylindole (DAPI, Abcam) was used for nuclear counterstain. The cytoskeleton was stained with phalloidin-Alexa 488 (Cell Signaling). Image acquisition was performed using a Leica DMRB microscope (Leica) Images were taken with a digital camera (Spot 32, Diagnostic Instruments) with the same exposure time for all pictures.

Confocal laser scanning microcopy was done with fixed cells on a Leica SP8 equipped with a white light laser (Superk Extreme EXW-9 NIM, NKT Photonics, Denmark) and a 405 nm laser diode (LASOS, VLK 0550 T01), using a 100x oil immersion objective with a numerical aperture of 1.4 (HC PL APO 100x/1.40 OIL CS2). The fluorescence of DAPI was excited at 405 nm and its emission was recorded in the spectral window of 420 – 480 nm. The pH sensor dye was excited at 405 nm and its emission was detected in the spectral window of 520 – 560 nm. The reference dye was excited at 560 nm and its emission was detected in the spectral window of 570 – 660 nm. All channels were sequentially excited to minimize spectral bleed through. The pixel size of the recorded images was 29.36 nm. A z-stack over the height of the cells with a step size of 0.3 µm was recorded. All images were acquired with the same imaging settings. Adjustments of the intensity histograms of the images to improve the visibility of the fluorescence signal were equally applied to all images.

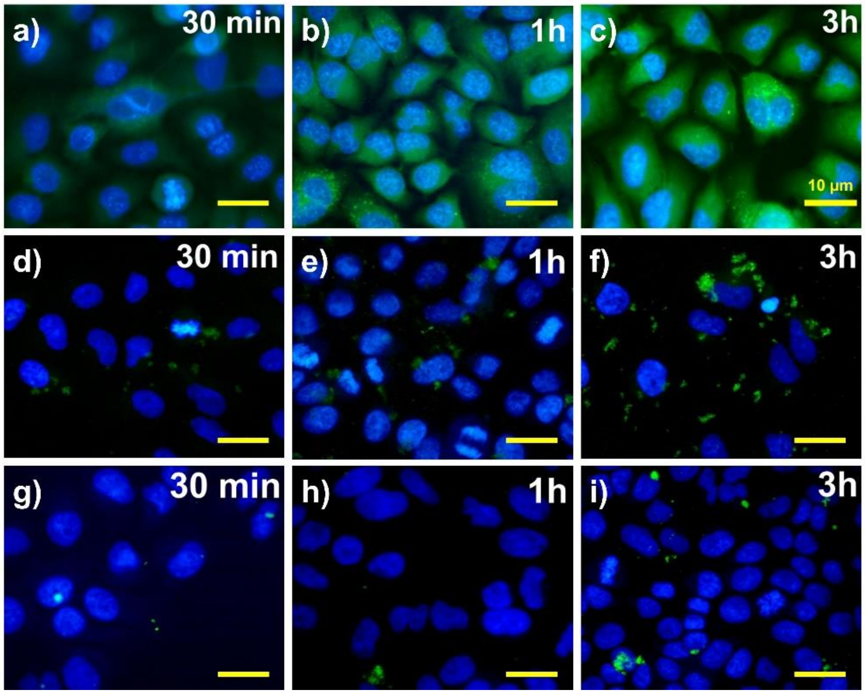

Figure S 37. Epifluorescence images at neutral pH of the uptake of free optical probe 3 (a-c), dye concentration 35 µg/mL, λem = 470 nm), and the pH nanosensors PSNP-NR-3 (d-f), particle concentration 100 µg/mL, λem = 470 nm) and SiO_2-RhB-3 (g-f), particle concentration 100 µg/mL, λem = 470 nm) by A549 cells measured after different incubation times, 30 min, 1 h and 3 h, respectively. Prior to the fluorescence microscopy studies, the cells were incubated alive, fixed with 4% paraformaldehyde (PFA), and then co-stained with DAPI (cell nuclei, blue channel: λem = 340/380 nm). Excitation was carried out with an Osram 50W/ACL1 Cz HBO Mercury vapor short-arc lamp. All images show a scale bar of 10 µm.

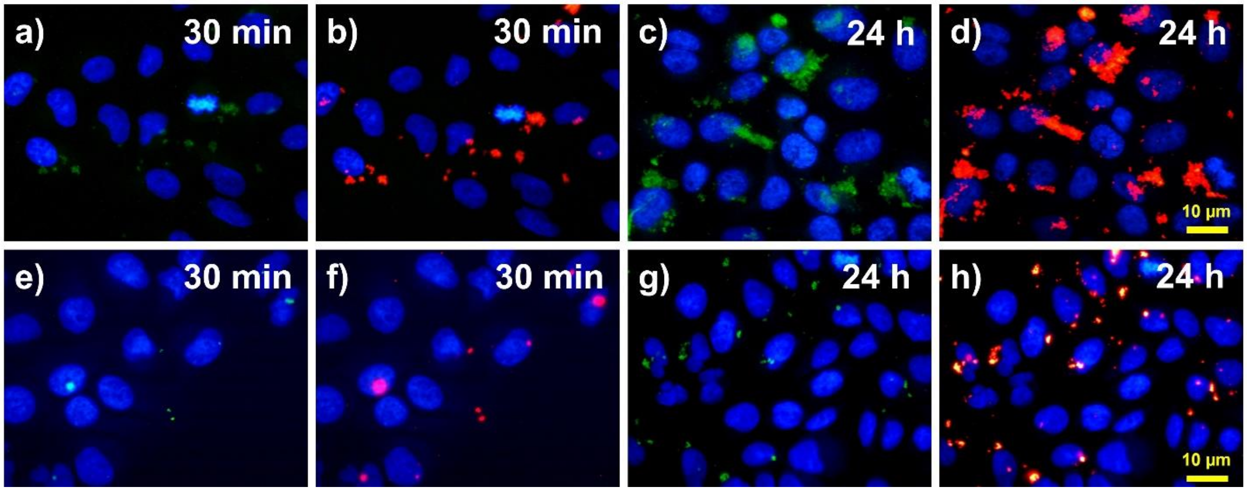

Figure S 38. Epifluorescence images of the pH nanosensors PSNP-NR-3 (a-d), particle concentration 100 µg/mL) and SiO_2-RhB-3 (e-h), particle concentration 100 µg/mL) by A549 cells measured after incubation times of 30 min, and 24 h, respectively. Prior to the fluorescence microscopy studies, the cells were incubated alive, fixed with 4% paraformaldehyde (PFA), and then co-stained with DAPI (cell nuclei, blue channel). Excitation was carried out with an Osram 50W/ACL1 Cz HBO Mercury vapor short-arc lamp and the fluorescence was monitored with emission filters set to λ_{Em} = 470 nm (green channel) and to λ_{Em} = 560 nm (red channel). For the detection of the fluorescence of DAPI Leica filter cube A (λ_{Em} = 340/380 nm (blue channel) was used. All images show a scale bar of 10 µm.

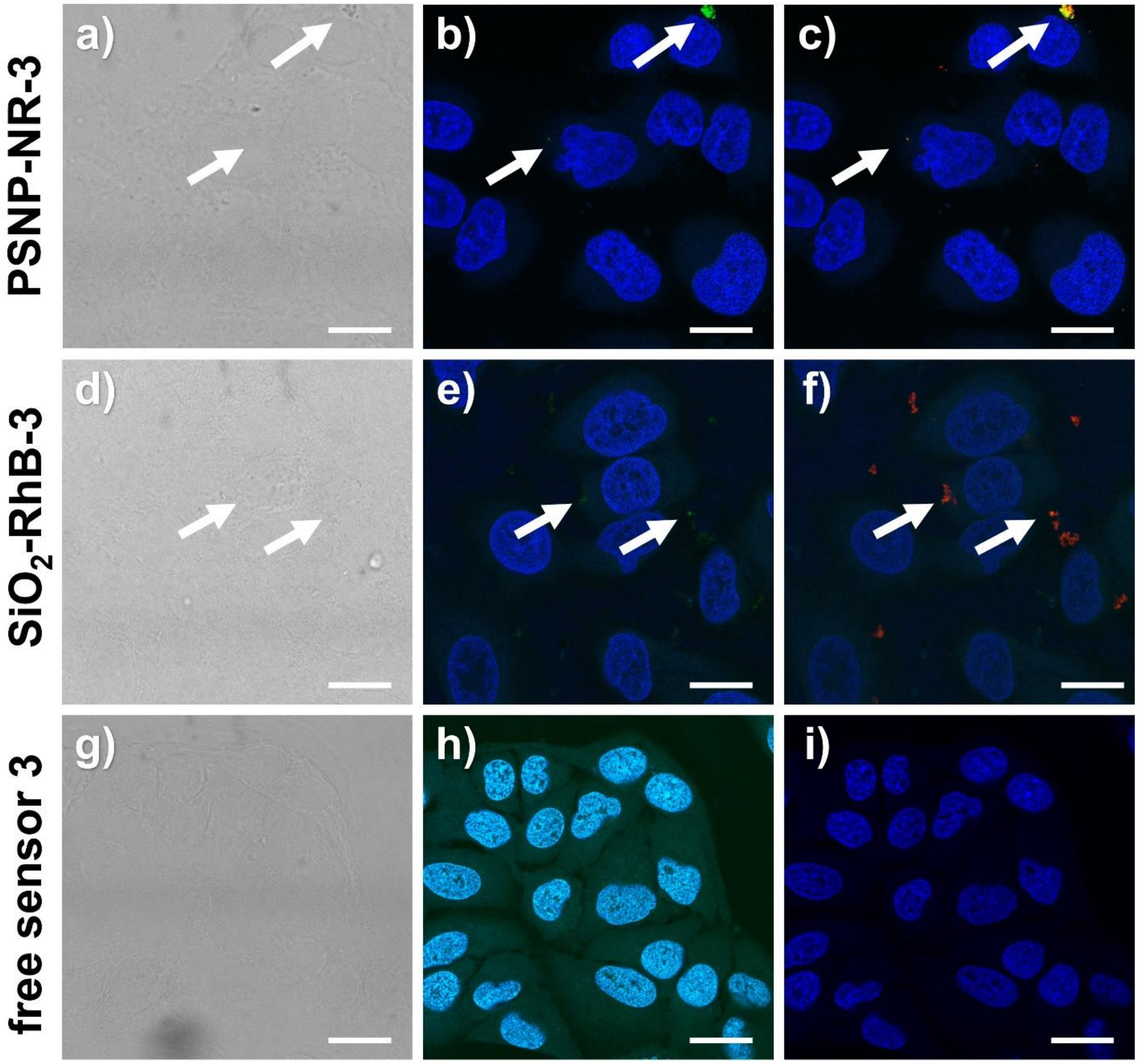

Figure S 39. Confocal laser scanning microscope (CLSM) images of fixed A549 cells (PFA) incubated for 30 min with PSNP-NR-**3** (a-c, particle concentration 100 µg/mL), SiO_2-RhB-**3** (d-f, particle concentration 100 µg/mL), and free pH sensor **3**, (g-I, sensor concentration 35 µg/mL) at pH 7.5 in PBS buffer. The nuclei were stained with DAPI. Transmitted light a), d), g); merged blue (DAPI) and green (pH sensor **3**) channels: b), e), h); and merged blue (DAPI), green (pH sensor **3**) and red (reference dyes, NR or RhB) channels: c), f), i). λ_{Ex} = 405 nm, λ_{Em1} = 420 - 480 nm and λ_{Em2} = 520 - 560 nm for DAPI and **3** respectively. and λ_{Ex} = 560 nm and λ_{Em} range 570 – 660 nm for the reference dyes. Scale bar 10 µM.

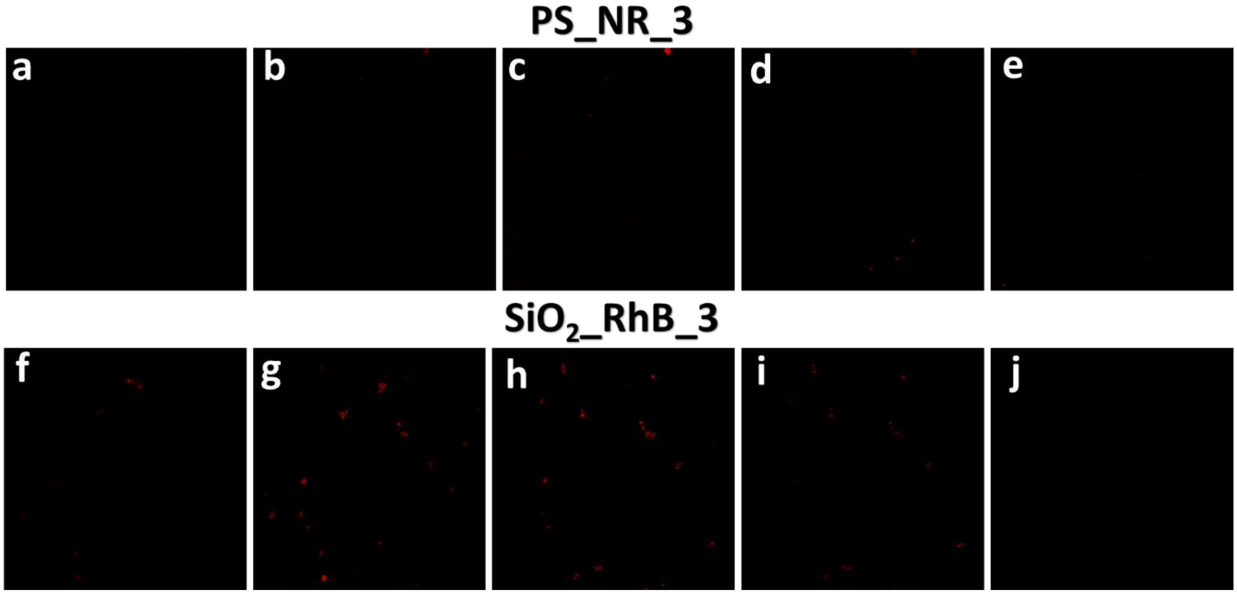

Figure S 40. CLSM z-stack images of A549 cells incubated 30 min and 3 h with PSNP-NR-**3** (a-e, particle concentration 100 µg/mL), and SiO_2-RhB-**3** (f-j, particle concentration 100 µg/mL) at pH 7.5 (z step size 1.5 µm; a -e and f-j top to bottom) λ_{Ex} = 560 nm, λ_{Em} of 570 - 660 nm).

6. References

1 Martin-Brown, S. A., Fu, Y., Saroja, G., Collinson, M. M. & Higgins, D. A. Single-Molecule Studies of Diffusion by Oligomer-Bound Dyes in Organically Modified Sol-Gel-Derived Silicate Films. *Anal. Chem* **77**, 486-494 (2005).

2 Jiang, Z. *et al.* Multifunctional Au@mSiO2/Rhodamine B Isothiocyanate Nanocomposites: Cell Imaging, Photocontrolled Drug Release, and Photothermal Therapy for Cancer Cells. *Small* **9**, 604-612, doi:https://doi.org/10.1002/smll.201201558 (2013).

3 Hennig, A. *et al.* Scope and Limitations of Surface Functional Group Quantification Methods: Exploratory Study with Poly(acrylic acid)-Grafted Micro- and Nanoparticles. *Journal of the American Chemical Society* **134**, 8268-8276, doi:10.1021/ja302649g (2012).

4 Srivastava, P. *et al.* Multicolor Polystyrene Nanosensors for the Monitoring of Acidic, Neutral, and Basic pH Values and Cellular Uptake Studies. *Analytical Chemistry* **94**, 9656-9664, doi:10.1021/acs.analchem.2c00944 (2022).

5 Lucien, F., Harper, K., Pelletier, P.-P., Volkov, L. & Dubois, C. M. Simultaneous pH Measurement in Endocytic and Cytosolic Compartments in Living Cells using Confocal Microscopy. *JoVE*, e51395, doi:doi:10.3791/51395 (2014).
